# Prevention of intraoperative hypothermia: a descriptive-exploratory study*

**DOI:** 10.1590/1518-8345.7694.4728

**Published:** 2025-11-10

**Authors:** Layze Braz de Oliveira, Vanessa de Brito Poveda, Rosana Aparecida Spadoti Dantas, Julio Cesar Ribeiro, Cristina Maria Galvão

**Affiliations:** 1 Centro Universitário UniFacid, Instituto de Educação Médica, Teresina, PI, Brazil. Centro Universitário UniFacid Instituto de Educação Médica PI Teresina Brazil; 2 Scholarship holder at the Coordenação de Aperfeiçoamento de Pessoal de Nível Superior (CAPES), Brazil. Scholarship holder at the Coordenação de Aperfeiçoamento de Pessoal de Nível Superior Brazil; 3 Universidade de São Paulo, Escola de Enfermagem de Ribeirão Preto, PAHO/WHO Collaborating Centre for Nursing Research Development, Ribeirão Preto, SP, Brazil. Universidade de São Paulo Escola de Enfermagem de Ribeirão Preto PAHO/WHO Collaborating Centre for Nursing Research Development SP Ribeirão Preto Brazil; 4 Universidade de Franca, Faculdade de Enfermagem, Franca, SP, Brazil. Universidade de Franca Faculdade de Enfermagem SP Franca Brazil; 5 Scholarship holder at the Conselho Nacional de Desenvolvimento Científico e Tecnológico (CNPq), Brazil. Scholarship holder at the Conselho Nacional de Desenvolvimento Científico e Tecnológico Brazil

**Keywords:** Perioperative Nursing, Hypothermia, Intraoperative Period, Body Temperature, Hospitals, Patient Safety

## Abstract

to analyze the measures implemented in practice for the prevention of hypothermia during the intraoperative period in public and private hospitals.

quantitative, descriptive-exploratory study. The sample consisted of 201 nurses, invited via social media, Facebook, Instagram, and email. Data collection was performed using an online form created on the Survey Monkey^®^ virtual platform. For analysis, descriptive statistics and association tests were applied to investigate the differences between the defined groups in relation to the study variables.

in both types of institutions, most participants were female and married/in a stable relationship. There was a significant difference between the types of hospitals in terms of operating room temperature control (p<0.001), patient body temperature monitoring (p=0.027), and use of active skin warming methods (p=0.009).

the forced-air warming was the active method most frequently used, and nursing staff were the professional category most often indicated as responsible for implementing prewarming and using active methods in the types of institutions investigated. There is still a need to increase the daily implementation of measures to prevent hypothermia, pointing out to managers the need for investment and training of human resources.

## Highlights

**(1)** Intraoperative hypothermia is a common event that can affect surgical patients. **(2)** Preventing hypothermia is a challenging practice for the entire intraoperative team. **(3)** Measuring body temperature is a recommended practice during the intraoperative period. **(4)** Prewarming is an important measure for maintaining normothermia.**(5)** The forced-air warming is an effective measure for preventing hypothermia.

## Introduction

Intraoperative hypothermia is commonly defined as a body temperature below 36°C and is mainly related to the use of anesthetic agents that cause changes in hypothalamic temperature regulation and body heat redistribution. Other factors that may contribute to this event include low ambient temperature, exposure of the patient’s organs or tissues, and the ambient temperature of intravenous solutions and irrigation fluids^(1-3)^.

There is a body of evidence in the literature indicating that intraoperative hypothermia is associated with cardiac events, surgical site infection, increased blood loss and transfusion requirements, decreased drug metabolism, thermal discomfort, increased length of stay in the post-anesthesia recovery room, and increased hospital costs^(1-5)^.

In clinical practice, this complication is common. In the United States, the incidence of hypothermia in the intraoperative period ranged from 20% to 70%^(3)^, and in Australia it was 42%^(4)^. In Brazil, its prevalence was 56.7%^(6)^.

In a systematic review with meta-analysis, the authors investigated the risk factors for hypothermia in the intraoperative period in adult patients. The sample consisted of 12 studies involving 15,010 patients. Age, body mass index, ambient temperature, systolic blood pressure and heart rate (in the preoperative period), long duration of anesthesia (> 2 hours) and administration of intravenous solutions (> 1,000 ml) were identified as risk factors^(7)^.

The main recommendations for the prevention of intraoperative hypothermia are aimed at monitoring and documenting the patient’s body temperature in the intraoperative period, the use of active methods to maintain normothermia (e.g., forced-air warming), including the implementation of prewarming before anesthetic induction when the patient’s temperature is below 36°C. The combination of passive methods (e.g., the use of heated cotton sheets) with active methods is also recommended, as well as assessing specific factors related to the patient (e.g., age) and those related to the surgical anesthetic procedure (e.g., type and duration of surgery and anesthesia). In addition, minimizing the patient’s exposure to the cold environment is one approach to reducing heat loss. Another relevant aspect is maintaining the ambient temperature, which is recommended to be at least 21°C^(2,5)^.

Despite the relevance of preventing intraoperative hypothermia pointed out in the literature, in clinical practice there are problems in recognizing the importance of this event by health professionals and health institution managers, monitoring the patient’s body temperature, and implementing active methods. In addition, nurses’ knowledge and practice regarding this issue are inadequate when compared to the clinical guidelines of scientific societies^(8-10)^.

In view of the above, this research was conducted to generate evidence to support clinical practice, since maintaining the patient’s body temperature and implementing effective measures to prevent hypothermia in the intraoperative period are current challenges faced by the team of health professionals involved in the care of surgical patients, especially nurses. In addition, it is worth noting the scarcity of studies on this issue in the national context, especially in institutions with different legal statuses. Thus, the specific objective was to analyze the measures implemented in practice to prevent hypothermia during the intraoperative period in public and private hospitals.

## Method

### Study design

This is a quantitative, descriptive-exploratory study.

### Setting

The data collection site was not determined because the research was conducted in a virtual environment, where potential participants could be included in the study regardless of their physical location within the country.

### Population and sample eligibility criteria

The target population of the study consisted of nurses working in operating rooms in Brazilian public and private hospitals. A non-probabilistic sample was formed of 201 nurses who met the following eligibility criteria: having an active employment relationship as a nurse, having worked in the operating room (OR) of the hospital institution for at least six months, regardless of the type of activities performed, and accessing the online form between January and August 2022. Seven nurses who completed the form in full were not included in the final sample because they worked as perfusionists (three) and in the sterilized material center (two), as well as two who were pursuing residency in surgical clinic. A nursing technician responded to the survey and was also not considered.

### Data collection

For the online recruitment of potential participants, different strategies were employed on social media platforms Facebook and Instagram. On Facebook, the group “Nursing in the operating room and sterile supply center” was identified, which has 1,800 members. In this group, potential participants were identified, i.e., nurses working in operating rooms, and the principal investigator promoted a post to the selected individuals through targeting, such as: place of work; people who liked the researcher’s page, as well as their friends and previously defined custom audiences.

When consulting the Instagram of the Brazilian Association of Operating Room Nurses, Anesthetic Recovery, and Material and Sterilization Center (SOBECC), for those people who listed in their profile higher education in nursing with experience in OR, the principal investigator sent an invitation to the profile of each professional identified on this social network.

SOBECC has a database of emails of nurses who work in intraoperative patient care. After approval by the board of this organization to use this database, the principal investigator sent the registered members the electronic link generated by the Survey Monkey^®^ virtual platform. Due to low participant adherence, two attempts were made to use this data collection strategy.

Potential participants were provided with an electronic link that allowed them to access the research invitation, the Free and Informed Consent Form (FICF), and the data collection form from January to August 2022.

### Data collection form and study variables

An online form was created on the Survey Monkey^®^ virtual platform, containing 28 questions divided into three sections: nine for characterizing nurses, 16 on measures for preventing hypothermia during surgery, and three on difficulties in preventing this complication. The questions were developed based on the literature^(2,11)^ and the intellectual production of the authors of this research on the problem investigated.

The dependent variable was the type of health institution (public or private). The independent variables were grouped by themes, namely: sociodemographic characterization (e.g., gender, age, marital status) and nurses’ training (e.g., type of complementary training), measures for the prevention of hypothermia (e.g., use of prewarming, active and passive methods), and difficulties in preventing this event during the intraoperative period (e.g., reasons for not monitoring the patient’s body temperature and the availability of medical devices).

### Data processing and analysis

The data were stored in Excel spreadsheets and subsequently transferred to IBM^®^ SPSS^®^ Statistics software version 25.

For data presentation, participants were divided into two groups: nurses working in public hospital ORs and those working in private institutions. Qualitative variables were described by the frequency (absolute and relative) of participant distribution among the defined categories, and quantitative variables were evaluated in terms of position (mean) and dispersion (standard deviation). Association tests (Pearson’s chi-square test or Fisher’s exact test) were applied to investigate the differences between the defined groups in relation to the variables measured for prewarming, measures for preventing hypothermia, and difficulties in preventing this event during the intraoperative period. For all analyses, the level of significance considered was 5% (α=0.05).

### Ethical aspects

The study was approved by the local Research Ethics Committee, under Opinion CAAE: 43126521.5.0000.5393. All participants granted informed consent online by selecting the option “I agree to participate in the study”. All ethical principles established in the legislation were met.

## Results

Table 1 shows the sociodemographic and educational variables of the study participants. In both types of hospitals, most participants were female, married/in a stable relationship, with a specialization and a single employment bond.

**Table 1-:** Sociodemographic and educational variables of nurses working in operating rooms in public and private hospitals. Ribeirão Preto, SP, Brazil, 2022

Variables	Public (n*=139)		Private (n*=62)		Total (n*=201)	
	n*	% ^†^	n*	% ^†^	n*	% ^†^
Gender						
Female	119	85.6	56	90.3	175	87.1
Male	20	14.4	6	9.7	26	12.9
Age group (years)						
<30	11	7.9	13	21.0	24	11.9
30–39	58	41.7	24	38.7	82	40.8
40–49	48	34.5	13	21.0	61	30.3
50–59	13	9.4	8	12.9	21	10.4
≥60	9	6.5	4	6.5	13	6.5
Marital status						
Single	46	33.1	23	37.1	69	34.3
Married/civil union	76	54.7	32	51.6	108	53.7
Divorced/separated/widowed	17	12.2	7	11.3	24	12.0
Length of education (years)						
<5	15	10.8	14	22.6	29	14.4
5–10	33	23.7	18	29.0	51	25.4
>10	91	65.5	30	48.4	121	60.2
Additional education						
Yes	122	87.8	55	88.7	177	88.1
No	17	12.2	7	11.3	24	11.9
Levels of additional education						
Residency	2	1.4	1	1.6	3	1.5
Specialization	86	61.9	47	75.8	133	66.2
Master’s degree	21	15.1	4	6.5	25	12.4
Doctorate	13	9.4	2	3.2	15	7.5
Not specified	-	-	1	1.6	1	0.5
None	17	12.2	7	11.3	24	11.9
Employment bond						
One	104	74.8	52	83.9	156	77.6
Two	35	25.2	10	16.1	45	22.4
Time working in the operating room (years)						
<5	43	30.9	23	37.1	66	32.8
5–10	34	24.5	18	29.0	52	25.9
>10	62	44.6	21	33.9	83	41.3

*n = Absolute number; ^†^% = Percentage

In public hospitals, the age of nurses ranged from 23 to 74 years, with an average of 40.9 years (standard deviation = 9.6), concentrating in the 30 to 49 age range (76.2%), with training time ranging from zero (six months) to 46 years (median 12 years), and operating room work experience ranging from zero (six months) to 40 years (median eight years). In private hospitals, the age of participants ranged from 23 to 72 years, with an average of 39.2 years (standard deviation = 10.9), with most in the 30 to 49 age range (59.7%), between one and 40 years since graduation (median 10 years), and with operating room work experience ranging from zero (six months) to 30 years (median six years and six months) (data not shown in table).

In the intraoperative period, with regard to operating room temperature control, the results showed that this practice was more frequent in private hospitals (88.7%) compared to public hospitals (58.3%), with a significant difference (p<0.001). Monitoring the patient’s body temperature was also more frequent in private institutions (35.4%) compared to public ones (29.4%) (p=0.027).

The axillary thermometer was the most used device for measuring body temperature in public institutions (53.5%) and the esophageal thermometer (34.5%) in private institutions, p=0.003. The anesthesiologist was the professional most often indicated as responsible for monitoring the patient’s body temperature in both types of hospitals, p=0.014.

Table 2 shows data on prewarming as a measure for preventing hypothermia.

Table 3 shows the measures taken to prevent hypothermia. Increasing the temperature of the operating room was one measure, and the results showed a significant difference between the types of hospitals (p=0.003), with this practice being performed more frequently in public hospitals. Regarding the use of active skin warming methods among the types of institutions, the results showed a significant difference (p=0.009). The forced-air warming was the active method most frequently indicated in both types of institutions.

Nursing was the professional category most frequently indicated for the implementation of active skin warming methods, 59.7% in public hospitals and 75.8% in private hospitals, with a significant difference (p=0.027).

Table 4 shows the difficulties encountered in preventing hypothermia.

**Table 2-:** Prewarming of patients before anesthetic induction, as reported by operating room nurses in public and private hospitals. Ribeirão Preto, SP, Brazil, 2022

Prewarming	Pubic (n*=139)		Private (n*=62)		p ^‡^
	n ^*^	% ^†^	n ^*^	% ^†^	
Performed in a surgical setting					0.008
Yes	98	70.5	55	88.7	
No	41	29.5	7	11.3	
Measures adopted ^§^					0.055
Passive method	39	28.1	22	35.5	
Active method	45	32.4	24	38.7	
Mixed method	12	8.6	7	11.3	
Never performed	41	29.5	7	11.3	
Professional responsible ^ǁ^					0.029
Anesthesiologist	16	11.5	6	9.7	
Nurse	57	41.0	33	53.2	
Both professional categories	19	13.7	13	21.0	
Never performed	41	29.5	7	11.3	

*n = Absolute number; ^†^% = Percentage; ^‡^Pearson’s chi-square test; ^§^Not reported: two for each type of hospital; ^ǁ^Not reported: six for public hospitals and three for private hospitals

**Table 3-:** Measures for the prevention of hypothermia in the intraoperative period, as reported by operating room nurses in public and private hospitals. Ribeirão Preto, SP, Brazil, 2022

Variables	Public (n*=139)		Private (n*=62)		p ^‡^
	n*	% ^†^	n*	% ^†^	
Increase the temperature of the operating room					0.003 ^§^
Yes	57	41.0	12	19.4	
No	82	59.0	50	80.6	
Use of passive skin warming method					0.212 ^§^
Yes	67	48.2	24	38.7	
No	72	51.8	38	61.3	
Use of active skin warming method					0.062 ^§^
Yes	77	55.4	43	69.4	
No	62	44.6	19	30.6	
Use of mixed method					0.873 ^§^
Yes	19	13.7	9	14.5	
No	120	86.3	53	85.5	
Infusion of heated solution intravenously					0.385 ^§^
Yes	67	48.2	34	54.8	
No	72	51.8	28	45.2	
Heated solution for cavity irrigation					0.285 ^§^
Yes	41	29.5	23	37.1	
No	98	70.5	39	62.9	
Active warming method is performed					0.009 ^ǁ^
Yes	113	81.3	59	95.2	
No	26	18.7	3	4.8	
Professional performing active warming method					
Anesthesiologist					0.067 ^§^
Yes	68	48.9	39	62.9	
No	71	51.1	23	37.1	
Surgeon					0.403 ^§^
Yes	7	5.0	5	8.1	
No	132	95.0	57	91.9	
Nurse					0.027 ^§^
Yes	83	59.7	47	75.8	
No	56	40.3	15	24.2	

*n = Absolute number; ^†^% = Percentage; ^‡^Significance level; ^§^Pearson’s chi-square test; ^ǁ^Fisher’s exact test

**Table 4-:** Difficulties in preventing hypothermia in the intraoperative period, as reported by operating room nurses in public and private hospitals. Ribeirão Preto, SP, Brazil, 2022

Variables	Public (n*=139)		Private (n*=62)		p ^‡^
	n*	% ^†^	n*	% ^†^	
	n*	% ^†^	n*	% ^†^	
Reasons for not monitoring body temperature					
Anesthesia team does not consider this practice relevant in the operating room					0.342 ^§^
Yes	33	23.7	11	17.7	
No	106	76.3	51	82.3	
Surgical team does not consider this practice relevant in the operating room					0.672 ^§^
Yes	13	9.4	7	11.3	
No	126	90.6	65	88.7	
Difficulties in implementing this practice among healthcare professionals					0.118 ^§^
Yes	39	28.1	11	17.7	
No	100	71.9	51	82.3	
Medical devices required are not sufficient for this practice					0.014 ^§^
Yes	43	30.9	9	14.5	
No	96	69.1	53	85.5	
Materials used for passive warming are sufficient for daily demand					0.096 ^ǁ^
Never	17	12.2	3	4.8	
Rarely	17	12.2	3	4.8	
Sometimes	18	12.9	5	8.1	
Often	33	23.8	19	30.6	
Always	54	38.9	32	51.7	
Difficulties in using active skin warming methods					
There are not enough medical devices for daily demand					0.080 ^§^
Yes	56	40.3	17	27.4	
No	83	59.7	45	72.6	
There are no medical devices for patients with risk factors					0.151 ^§^
Yes	21	15.1	15	24.2	
No	118	84.9	47	75.8	
There are enough medical devices for daily demand, but professionals do not consider the practice relevant					0.352 ^ǁ^
Yes	11	7.9	2	3.2	
No	128	92.1	60	96.8	
Difficulty coordinating among healthcare professionals involved in care					0.159 ^§^
Yes	40	28.8	12	19.4	
No	99	71.2	50	80.6	

*n = Absolute number; ^†^% = Percentage; ^‡^Significance level; ^§^Pearson’s chi-square test; ^ǁ^Fisher’s exact test; Note: the variables reasons for not monitoring body temperature and difficulties in using active skin warming methods are multiple choice, so the total exceeds the sample value

## Discussion

As already mentioned, intraoperative hypothermia is associated with various complications. The literature contains a body of evidence and clinical guidelines from different scientific societies, indicating the importance of implementing measures to prevent this event. However, in clinical practice, the occurrence of hypothermia is still high^(12-13)^.

In the present study, temperature control in the operating room and monitoring of the patient’s body temperature were more frequent practices in private hospitals. In addition, the axillary thermometer was the most commonly used device to measure body temperature in public institutions, and the esophageal thermometer in private institutions (all results with statistical difference).

Operating room temperature is considered a risk factor for intraoperative hypothermia. Currently, the recommended parameter is ≥ 21ºC^(7,10,14)^. In the intraoperative period, in two observational studies, the authors investigated body temperature monitoring practices, and in one study (n=1,690 patients), the monitoring standard was low (n=687; 40.7%) in the preoperative period (within one hour before the patient was transferred to the operating room) and in the intraoperative period, only 22.4% (n=379) of participants had their temperature monitored ≥ once, while 94% (n=1,515) were assessed upon admission for postoperative care. Ear canal (infrared) and oral cavity thermometers were the most frequently used in all phases^(15)^.

In the other study, with the participation of the intraoperative team (nurses, anesthetists, and anesthesia technicians), the results showed that admission to surgery (n=384, 76%) was the most routine phase for temperature monitoring; in the intraoperative period, 56% of respondents (n=281) answered that temperature monitoring was performed continuously in their workplace, and 93% (n=472) reported temperature monitoring upon arrival in the post-anesthesia recovery room. Respondents identified ear canal devices as the most commonly used devices both preoperatively (n=239, 47%) and postoperatively (n=286, 57%). In the intraoperative period, nasopharyngeal devices were identified as the most frequently used (n=331, 66%), followed by esophageal (n=229, 45%) and urinary bladder (n=202, 40%) devices^(12)^.

In a national longitudinal study, during the intraoperative period, the authors investigated the reliability of body temperature measurements using three methods (peripheral infrared temporal thermometer, central skin thermometer, Zero-Heat-Flux, and esophageal or nasopharyngeal thermometer). The sample consisted of 99 patients undergoing elective abdominal cancer surgery. Based on the results, the researchers did not recommend the use of infrared temporal thermometers to measure body temperature. The two central thermometers tested were equivalent in detecting hypothermia^(16)^.

Given the above, the use of axillary thermometers to measure patients’ body temperature is a practice that should be reconsidered, especially in public institutions.

Prewarming as a measure to prevent hypothermia was also performed more frequently in private hospitals (results with a significant difference). There is a body of evidence in the literature that proves the effectiveness of this measure in maintaining normothermia in surgical patients. In a randomized clinical trial, the specific objective was to evaluate the effect of different prewarming periods on body temperature in patients undergoing transurethral resection (bladder or prostate) under general anesthesia. The sample consisted of 297 patients randomized into four groups, namely: control group (n=76), 15-minute intervention group (n=74), 30-minute intervention group (n=73), and 45-minute intervention group (n=74). Prewarming was performed in the pre-anesthesia room using a forced-air warming. During the intraoperative period, all study participants also received forced-air warming. Core temperature was significantly higher in the 15- and 30-minute intervention groups (36.8°C, p=0.004; 36.7°C, p=0.041, respectively). In addition, at the end of surgery, body temperature was significantly lower in the control group (35.8°C) than in the three groups that received prewarming (p<0.001)^(17)^.

In another randomized clinical trial, the authors investigated the effect of prewarming on the body temperature of patients undergoing abdominal surgery (open surgical approach) and their level of thermal comfort. Patients were randomized into three groups: control group (n=33; prewarming with blanket and cotton sheet); intervention group 1 (n=33, prewarming with forced-air warming for 20 minutes) and intervention group 2 (n=33, prewarming with forced-air warming for 30 minutes). During the intraoperative period, all patients were warmed using forced-air warming, in addition to warming infusion and irrigation fluids using specific equipment. Thermal comfort was determined by patient self-reporting in the pre- and post-anesthetic periods. Intervention 2 showed the best results in relation to temperature, with the lowest average number of episodes of temperature below 36°C during the intraoperative period and greater thermal comfort reported by patients^(18)^.

In a systematic review with meta-analysis, the authors investigated the effect of prewarming (before anesthetic induction) on body temperature, including 27 randomized clinical trials. The sample size of the studies ranged from 16 to 416 patients. Different active methods of skin warming were used in the studies (e.g., forced-air warming system, electric blanket, carbon fiber blanket, among others), as well as different warming times (10 to 120 minutes). The results showed that prewarming is an effective measure for maintaining intraoperative normothermia^(3)^.

In the present study, in both types of hospitals, the forced-air warming was the most frequently indicated active method. In a systematic review with meta-analysis, the authors investigated the effectiveness of different active warming systems (forced-air warming system, heated water circulation system, and electric blanket) in preventing hypothermia and shivering when applied to specific areas of the human body. The review included 24 randomized clinical trials involving 1,119 adult patients undergoing abdominal surgery. Patients in the control group underwent passive warming methods. The primary outcome measured was core body temperature at 60 and 120 minutes after anesthetic induction. The different disposable devices (upper limbs, lower limbs, or whole body placed on the operating table) of the forced-air system were investigated. The results of the network meta-analysis showed that the forced-air system (whole-body device) effectively raised core body temperature and prevented shivering in recovering patients^(13)^.

In another systematic review with meta-analysis, the researchers also analyzed the effect of using the forced-air warming and heated intravenous fluids (combined strategy) in patients undergoing cesarean section with regional anesthesia. The review evaluated nine randomized clinical trials involving 595 women. The combined strategy reduced the incidence of hypothermia and shivering, with an improvement in the maternal comfort score (results with a significant difference)^(19)^.

In a randomized clinical trial, the authors investigated the effect of a forced-air warming placed on different parts of the body. Thus, 537 patients undergoing open abdominal surgery were randomized into groups A (disposable device on upper limbs), B (lower limbs), and C (entire body placed on the operating table). Intraoperative hypothermia occurred in 51.4% of patients in group B, 37.6% in group A, and 34.1% in group C (p=0.002). The results showed that the use of the forced-air warming, placed on the upper limbs or entire body, reduced hypothermia and prolonged the time of maintaining the core temperature above 36°C before the onset of this event^(20)^.

Nurses were the professional category most frequently responsible for implementing prewarming and active warming methods in both types of hospitals (results with significant difference). Nurses play an important role in preventing complications in surgical patients, including the prevention of hypothermia. However, maintaining normothermia is the responsibility of the entire intraoperative team (surgeons, anesthesiologists, and nursing staff)^(10,21)^.

In the present study, among the reasons for not monitoring body temperature during the intraoperative period, there was only a significant difference between public and private hospitals, the reason being that the necessary medical devices are not sufficient for the adoption of this practice.

In a qualitative study, the researchers applied an online questionnaire to identify the main factors that prevent anesthetists from performing adequate body temperature management. The sample consisted of 195 participants, of whom 81% (n=158) responded that they were unaware of the clinical guidelines for body temperature monitoring, and 95.4% (n=186) stated that they sometimes neglected to take measures to prevent hypothermia, especially during emergency surgery and patient transfers. Two categories (environmental and insufficient resources) that hindered temperature monitoring were reported by 99% (n=193) of anesthesiologists. Only 12.8% (n=25) reported that they frequently monitored body temperature and did so as a daily routine^(22)^.

In another qualitative study, the objective was to reveal the perceptions of operating room nurses (n=17) about intraoperative hypothermia, as well as their experiences and recommendations for its prevention. The absence of equipment, specifically the forced-air warming, due to inadequate availability was also a barrier pointed out in the management of this event. The nurses reported that when observing a patient with cold, alternative measures were implemented, such as the use of radiant heaters and electric blankets, raising the room temperature, or using additional blankets (e.g., heated cotton sheets). In addition, the warming of irrigation and intravenous fluids, as well as blood and blood products, were also measures reported by the research participants. The authors concluded that the different measures mentioned are due to the lack of institutional standardization, the inadequacy of the active heating system, and the lack of care protocols to guide nurses in planning and implementing measures to prevent hypothermia^(23)^.

Despite the body of evidence on the importance of preventing intraoperative hypothermia through the implementation of effective measures, there are still gaps between knowledge production and clinical practice. This scenario may occur due to insufficient knowledge of this problem by health professionals, as well as their low adherence to the clinical guidelines of scientific societies. In a cross-sectional study involving nurses and anesthesia technicians (n=219), the average knowledge score of healthcare professionals regarding hypothermia management was 13.78, meaning that 79.5% of participants had a moderate level (≤10 points: low; 11-20 points: moderate, ≥21 points: high)^(10)^.

In a cross-sectional study conducted in China, the specific objective was to investigate the knowledge, attitudes, and practices of healthcare professionals regarding hypothermia prevention. The sample consisted of 14 surgeons, 29 anesthesiologists, and 170 nurses. The mean scores for knowledge, attitudes, and practices were 5.36 (total score of 12), 47.54 (total score of 55), and 31.57 (total score of 40), respectively. Most participants had positive attitudes and acceptable practices; however, knowledge about the prevention of this event was inadequate. The authors highlighted the need for training programs and standardized protocols to improve adherence to guidelines for the prevention of intraoperative hypothermia^(24)^.

In another cross-sectional study, the sample consisted only of nurses (n=413). The results showed that 59.1% of participants had good knowledge, and most (50.4%) had good practices regarding the prevention of intraoperative hypothermia. Factors associated with nurses’ knowledge included being male, having a bachelor’s degree, having a master’s degree, and participating in training. Factors associated with clinical practice were working in the post-anesthesia recovery room or intensive care unit, participating in training, being satisfied with the job, and having good knowledge. The authors concluded that hospital managers should invest in strategies (e.g., training programs) to increase knowledge and implementation of measures for the prevention of hypothermia^(8)^.

In a national descriptive-exploratory study, the objective was to evaluate the knowledge and interventions of the nursing team regarding intraoperative hypothermia. Seventy-seven members of the nursing team (nurses and technicians) working in the operating room and post-anesthesia recovery room participated. Of the results shown, 45.7% of participants reported the importance of monitoring body temperature during the pre-, intra-, and postoperative periods, with the following practices being the most well-known: use of a heater (35.5%), use of a forced-air warming (28.3%), heated saline solution (16.9%), and blankets (15.1%). Warming the patient (58.6%) and administering heated saline solution (31.5%) were the two measures that obtained the highest frequencies in the operating room. The authors concluded that there are still gaps in knowledge and practices for the prevention of hypothermia, requiring the planning of educational actions to improve the quality of care provided to surgical patients^(25)^.

In a descriptive study, the researchers also investigated knowledge and practices for the prevention of hypothermia. The sample consisted of 122 nurses. The data collection script was based on the clinical guidelines of the Association of Intraoperative Registered Nurses; National Institute for Health and Care Excellence; Turkish Society of Anesthesiology and Reanimation Guideline. The results showed that 80.3% of nurses indicated the use of a passive method, 49.1% the use of an active method (heated air), and 36.9% the warming of blood and fluids before administration. In conclusion, the authors pointed out that nurses did not use the methods recommended by scientific societies for the prevention of intraoperative hypothermia^(9)^.

Currently, there are general recommendations in the literature, based on evidence, which can guide the development and implementation of care protocols for the prevention of intraoperative hypothermia. For all phases of the intraoperative period (pre, intra, and post), the main principles are monitoring core body temperature, warming the patient by active methods, and reducing exposure to cold. Temperature monitoring should be performed for all patients and, whenever possible, continuously. Active warming methods should be used to maintain the patient’s temperature above 36°C and their thermal comfort (a forced-air warming is recommended). Exposure to cold should be minimized by keeping the patient covered whenever possible, avoiding heat loss through radiation, conduction, convection, and evaporation. During the intraoperative period, the room temperature should be maintained at least 21°C, and intravenous fluid warming should be implemented^(5,26)^.

In the intraoperative period, the development of a care protocol for the prevention of hypothermia can assist healthcare professionals in making decisions about best practices for monitoring body temperature and implementing effective measures to reduce this complication in clinical practice. However, the protocol must be developed in line with an understanding of the main difficulties experienced in the health service in maintaining patient normothermia, for example, the insufficient number of devices for both body temperature monitoring and the use of active skin warming systems. In addition, the reduced awareness of managers about the problem may make the necessary financial investments in technology unfeasible^(4-5,10,27)^.

Given the results shown, it can be inferred that the measures reported by nurses for the prevention of hypothermia are in line with what is recommended in the literature. On the other hand, there is still a need to increase the daily implementation of these measures, especially in public hospitals, since the percentages related to monitoring the patient’s body temperature, performing prewarming, and using active skin warming methods were higher in private institutions (results with a significant difference). In addition, there is a need for investment and training of human resources for the effective management of this common event in the intraoperative period.

The study generated a body of evidence that can support clinical practice and aid decision-making by health professionals involved in the care of surgical patients, especially intraoperative nurses. The prevention of hypothermia can reduce the complications associated with this event, improve patient outcomes, and, above all, increase good practices in health care.

Regarding the limitations of the research, one related to the type of study stands out, that is, the data presented and discussed were reported by the participants. Although the principal investigator systematically promoted the invitation to nurses via social media and email, the sample size could have been larger, which can be characterized as low respondent adherence.

## Conclusion

The forced-air warming was the active method most frequently used, and nursing was the professional category most often indicated as responsible for implementing prewarming and using active methods in public and private hospitals.

During the intraoperative period, maintaining the patient’s normothermia is still a challenge, as it requires joint work between surgeons, anesthetists, and nursing staff. In addition to this situation, healthcare institutions need to invest in training healthcare professionals involved in surgical patient care, acquiring appropriate medical devices for monitoring body temperature, and purchasing effective skin warming equipment.

## Data Availability

The dataset that supports the findings of this study is not publicly available.
