# Supplementary material for: Prevention of intraoperative hypothermia: a descriptive-exploratory study
Source: Rev Lat Am Enfermagem. 2025 Nov 10;33:e4728. doi: 10.1590/1518-8345.7694.4728 (PMC12610928; doi:10.1590/1518-8345.7694.4728)
Supplement: Translation

# Translation: Prevenção da hipotermia no período intraoperatório: estudo descritivo-exploratório*

**DOI:** 10.1590/1518-8345.7694.4729

**Authors:** Layze Braz de Oliveira, Vanessa de Brito Poveda, Rosana Aparecida Spadoti Dantas, Julio Cesar Ribeiro, Cristina Maria Galvão

**Affiliations:** 1 Centro Universitário UniFacid, Instituto de Educação Médica, Teresina, PI, Brasil. Centro Universitário UniFacid Instituto de Educação Médica PI Teresina Brasil; 2 Bolsista da Coordenação de Aperfeiçoamento de Pessoal de Nível Superior (CAPES), Brasil. Bolsista da Coordenação de Aperfeiçoamento de Pessoal de Nível Superior Brasil; 3 Universidade de São Paulo, Escola de Enfermagem de Ribeirão Preto, Centro Colaborador da OPAS/OMS para o Desenvolvimento da Pesquisa em Enfermagem, Ribeirão Preto, SP, Brasil. Universidade de São Paulo Escola de Enfermagem de Ribeirão Preto Centro Colaborador da OPAS/OMS para o Desenvolvimento da Pesquisa em Enfermagem SP Ribeirão Preto Brasil; 4 Universidade de Franca, Faculdade de Enfermagem, Franca, SP, Brasil. Universidade de Franca Faculdade de Enfermagem SP Franca Brasil; 5 Bolsista do Conselho Nacional de Desenvolvimento Científico e Tecnológico (CNPq), Brasil. Bolsista do Conselho Nacional de Desenvolvimento Científico e Tecnológico Brasil

**Keywords:** Enfermagem Perioperatória, Hipotermia, Período Intraoperatório, Temperatura Corporal, Hospitais, Segurança do Paciente

## Abstract

analisar as medidas implementadas na prática para a prevenção da hipotermia, no período intraoperatório, em hospitais públicos e privados.

estudo quantitativo, descritivo-exploratório. A amostra foi composta por 201 enfermeiros, convidados via redes sociais, *Facebook* e *Instagram* e correio eletrônico. A coleta de dados foi realizada por meio de formulário *online* criado na plataforma virtual *Survey Monkey*^®^. Para análise, estatística descritiva e testes de associação foram aplicados para investigar as diferenças entre os grupos delimitados em relação às variáveis do estudo.

em ambos os tipos de instituições, a maioria dos participantes era do sexo feminino e casada/união estável. Houve diferença significante entre os tipos de hospitais no controle da temperatura da sala de operação (p<0,001), monitoramento da temperatura corporal do paciente (p=0,027) e uso de método ativo de aquecimento cutâneo (p=0,009).

o sistema de ar forçado aquecido foi o método ativo que obteve frequência maior e, a enfermagem, a categoria profissional mais indicada como responsável pela implementação do pré-aquecimento e uso de método ativo, nos tipos de instituições investigadas. Ainda existe a necessidade de incrementar a realização diária de medidas para a prevenção da hipotermia, apontando aos gestores a exigência de investimentos e qualificação de recursos humanos.

## Destaques

**(1)** Hipotermia perioperatória é evento comum que pode acometer o paciente cirúrgico. **(2)** A prevenção de hipotermia é prática desafiadora para toda a equipe perioperatória. **(3)** A mensuração da temperatura corporal é prática recomendada no período intraoperatório. **(4)** O pré-aquecimento é medida relevante para a manutenção da normotermia. **(5)** O sistema de ar forçado aquecido é medida efetiva para prevenção de hipotermia.

## Introdução

A hipotermia perioperatória é comumente definida como temperatura corporal abaixo de 36°C e está relacionada, principalmente, ao uso de agentes anestésicos que acarretam alterações na regulação hipotalâmica da temperatura e na redistribuição de calor corporal. Outros fatores que podem contribuir para tal evento incluem a baixa temperatura ambiente, exposição de órgãos ou tecidos do paciente, temperatura ambiente de soluções endovenosas e de fluidos de irrigação^(1-3)^.

Na literatura, existe corpo de evidências indicando que a hipotermia perioperatória está associada a eventos cardíacos, infecção de sítio cirúrgico, aumento de perda sanguínea e da necessidade de transfusão, diminuição do metabolismo de medicamentos, desconforto térmico, aumento da permanência na sala de recuperação pós-anestésica e dos custos hospitalares^(1-5)^.

Na prática clínica, a ocorrência desta complicação é comum. Nos Estados Unidos da América, no perioperatório, a incidência da hipotermia variou entre 20% e 70%^(3)^ e, na Austrália, a incidência foi de 42%^(4)^. No Brasil, a sua prevalência foi de 56,7%^(6)^.

Em revisão sistemática com metanálise, os autores investigaram os fatores de risco para hipotermia no período intraoperatório, em pacientes adultos. A amostra foi composta de 12 pesquisas envolvendo 15.010 pacientes. A idade, o Índice de Massa Corporal, a temperatura ambiente, pressão arterial sistólica e frequência cardíaca (no período pré-operatório), duração longa da anestesia (> 2 horas) e administração de soluções endovenosas (> 1.000 ml) foram apontados como fatores de risco^(7)^.

As principais recomendações para a prevenção da hipotermia perioperatória estão direcionadas para a monitorização e documentação da temperatura corporal do paciente no perioperatório, o uso de métodos ativos para a manutenção da normotermia (por exemplo, o sistema de ar forçado aquecido), inclusive para a implementação do pré-aquecimento, antes da indução anestésica, quando a temperatura do paciente estiver abaixo de 36^o^C. A combinação de métodos passivos (por exemplo, o uso de lençol de algodão aquecido) com ativos também é recomendada, bem como avaliar os fatores específicos relacionados ao paciente (por exemplo, a idade) e aqueles relacionados ao procedimento anestésico cirúrgico (por exemplo, tipo e duração da cirurgia e da anestesia). Além disso, minimizar a exposição do paciente ao ambiente frio é uma abordagem para reduzir a perda de calor. Outro aspecto relevante é manter a temperatura ambiente, sendo recomendado pelo menos a 21^o^C^(2,5)^.

Apesar da relevância da prevenção da hipotermia perioperatória apontada pela literatura, na prática clínica há problemas no reconhecimento da importância deste evento pelos profissionais de saúde e gestores de instituições de saúde, monitoramento da temperatura corporal do paciente e na implementação de métodos ativos. Além disso, o conhecimento e a prática dos enfermeiros sobre a problemática são inadequados na comparação com as diretrizes clínicas (*guidelines*) de sociedades científicas^(8-10)^.

Diante do exposto, a presente pesquisa foi conduzida para gerar corpo de evidências para subsidiar a prática clínica, uma vez que a manutenção da temperatura corporal do paciente e a implementação de medidas efetivas para a prevenção da hipotermia, no perioperatório, são desafios atuais enfrentados pela equipe de profissionais de saúde envolvida no cuidado do paciente cirúrgico, em especial o enfermeiro. Além disso, ressalta-se a escassez de estudos sobre a problemática no cenário nacional, com destaque para instituições com natureza jurídica diferente. Assim, o objetivo delimitado foi analisar as medidas implementadas na prática para a prevenção da hipotermia, no período intraoperatório, em hospitais públicos e privados.

## Método

### Delineamento do estudo

Trata-se de estudo quantitativo, descritivo-exploratório.

### Local

O local de coleta de dados não foi determinado por se tratar de pesquisa realizada em ambiente virtual cujos potenciais participantes poderiam ser inseridos no estudo independentemente da sua localização física no território nacional.

### População e critérios de elegibilidade da amostra

 A população-alvo do estudo era composta por enfermeiros que atuavam em centros cirúrgicos de hospitais brasileiros, públicos e privados. Uma amostra não probabilística foi formada por 201 enfermeiros que atenderam aos seguintes critérios de elegibilidade: ter vínculo trabalhista ativo como enfermeiro, estar atuando no centro cirúrgico (CC) da instituição hospitalar há, no mínimo, seis meses, sem distinção do tipo de atividades desempenhadas, acessar o formulário *online* no período de janeiro até agosto de 2022. Sete enfermeiros que preencheram integralmente o formulário não compuseram a amostra final por atuarem como perfusionistas (três) e na central de material esterilizado (dois), bem como dois cursavam residência em clínica cirúrgica. Um técnico de enfermagem respondeu à pesquisa e também não foi considerado. 

### Coleta de dados

Para o recrutamento *online* dos potenciais participantes, diferentes estratégias nas redes sociais *Facebook e Instagram* foram empregadas. No *Facebook*, o grupo de “Enfermagem em centro cirúrgico e centro de material esterilizado” foi identificado, possuindo 1,8 mil membros. No referido grupo, os potenciais participantes foram identificados, ou seja, enfermeiros que atuavam em CC, e o pesquisador principal impulsionou um post para as pessoas selecionadas por meio de direcionamentos, tais como: local de atuação no trabalho; pessoas que curtiram a página do pesquisador, bem como amigos dessas e públicos personalizados definidos anteriormente.

Ao consultar o *Instagram* da Associação Brasileira de Enfermeiros de Centro Cirúrgico, Recuperação Anestésica e Centro de Material e Esterilização (SOBECC), para aquelas pessoas que discriminaram no seu perfil formação superior em enfermagem com atuação em CC, o pesquisador principal encaminhou um convite no perfil de cada profissional identificado nesta rede social.

A SOBECC tem um banco de e-mails de enfermeiros que atuam no cuidado do paciente no perioperatório. Após aprovação pela diretoria da organização, no uso deste banco, o pesquisador principal encaminhou, para os membros cadastrados, o link eletrônico gerado pela plataforma virtual *Survey Monkey*^®^. Devido à adesão baixa dos participantes, duas tentativas foram realizadas no emprego dessa estratégia de coleta de dados.

Aos potenciais participantes identificados, disponibilizou-se um *link* eletrônico que permitia o acesso, no período de janeiro a agosto de 2022, ao convite da pesquisa, ao Termo de Consentimento Livre e Esclarecido (TCLE) e ao formulário de coleta de dados.

### Formulário de coleta de dados e variáveis do estudo

Um formulário *online* foi criado na plataforma virtual *Survey Monkey*^®^, contendo 28 questões, divididas em três seções: nove para caracterização dos enfermeiros, 16 sobre as medidas para a prevenção de hipotermia no período intraoperatório e três sobre as dificuldades para a prevenção desta complicação. As questões foram elaboradas pautadas na literatura^(2,11)^ e na produção intelectual dos autores da presente pesquisa sobre a problemática investigada.

A variável dependente foi “tipo de instituição de saúde” (pública ou privada). As variáveis independentes foram agrupadas por temas, a saber: caracterização sociodemográfica (por exemplo: sexo, idade, estado civil) e de formação dos enfermeiros (por exemplo: tipo de formação complementar), medidas para a prevenção da hipotermia (por exemplo: uso de pré-aquecimento, métodos ativos e passivos) e dificuldades para a prevenção desse evento no período intraoperatório (por exemplo: motivos para não realizar a monitorização da temperatura corporal do paciente e a disponibilidade de dispositivos médicos).

### Tratamento e análise dos dados

Os dados foram armazenados em planilhas eletrônicas no formato *Excel* e, posteriormente, transferidos para o *software* IBM^®^*SPSS*^®^*Statistics* versão 25.

Para a apresentação dos dados, os participantes foram divididos em dois grupos, a saber: enfermeiros com atuação no CC de hospitais públicos e aqueles com atuação em instituições privadas. As variáveis qualitativas foram descritas pela frequência (absoluta e relativa) de distribuição dos participantes entre as categorias delimitadas e as variáveis quantitativas foram avaliadas quanto à medida de posição (média) e dispersão (desvio-padrão). Os testes de associação (teste Qui-quadrado de Pearson ou teste exato de Fisher) foram aplicados para investigar as diferenças entres os grupos delimitados em relação às variáveis medidas de pré-aquecimento, medidas para prevenção da hipotermia e as dificuldades para prevenção deste evento no período intraoperatório. Para todas as análises, o nível de significância considerado foi de 5% (α=0,05).

### Aspectos éticos

O estudo foi aprovado pelo Comitê de Ética e Pesquisa local, sob Parecer CAAE: 43126521.5.0000.5393. Todos os participantes concederam o TCLE pela modalidade *online*, por meio da seleção da opção “Eu aceito participar da pesquisa”. Todos os preceitos éticos estabelecidos na legislação foram atendidos.

## Resultados

Na Tabela 1, as variáveis sociodemográficas e de formação dos participantes do estudo foram apresentadas. Em ambos os tipos de hospital, a maioria dos participantes era do sexo feminino, casada/união estável, com especialização e um único vínculo empregatício.

**Tabela 1-:** Variáveis sociodemográficas e de formação dos enfermeiros atuantes em centro cirúrgico de hospitais públicos e privados. Ribeirão Preto, SP, Brasil, 2022

Variáveis	Público (n*=139)		Privado (n*=62)		Total (n*=201)	
	n*	% ^†^	n*	% ^†^	n*	% ^†^
Sexo						
Feminino	119	85,6	56	90,3	175	87,1
Masculino	20	14,4	6	9,7	26	12,9
Faixa etária (anos)						
<30	11	7,9	13	21,0	24	11,9
30 – 39	58	41,7	24	38,7	82	40,8
40 – 49	48	34,5	13	21,0	61	30,3
50 – 59	13	9,4	8	12,9	21	10,4
≥60	9	6,5	4	6,5	13	6,5
Estado civil						
Solteiro	46	33,1	23	37,1	69	34,3
Casado/união estável	76	54,7	32	51,6	108	53,7
Divorciado/separado/viúvo	17	12,2	7	11,3	24	12,0
Tempo de formação (anos)						
<5	15	10,8	14	22,6	29	14,4
5 – 10	33	23,7	18	29,0	51	25,4
>10	91	65,5	30	48,4	121	60,2
Formação complementar						
Sim	122	87,8	55	88,7	177	88,1
Não	17	12,2	7	11,3	24	11,9
Níveis de formação complementar						
Residência	2	1,4	1	1,6	3	1,5
Especialização	86	61,9	47	75,8	133	66,2
Mestrado	21	15,1	4	6,5	25	12,4
Doutorado	13	9,4	2	3,2	15	7,5
Não especificou	-	-	1	1,6	1	0,5
Não possui	17	12,2	7	11,3	24	11,9
Vínculos empregatícios						
Um	104	74,8	52	83,9	156	77,6
Dois	35	25,2	10	16,1	45	22,4
Tempo de trabalho no centro cirúrgico (anos)						
<5	43	30,9	23	37,1	66	32,8
5 – 10	34	24,5	18	29,0	52	25,9
>10	62	44,6	21	33,9	83	41,3

*n = Número absoluto; ^†^% = Porcentagem

Nos hospitais públicos, a idade dos enfermeiros variou entre 23 e 74 anos, com uma média de 40,9 anos (desvio-padrão=9,6), concentrando-se na faixa entre 30 e 49 anos (76,2%), com tempo de formação de zero (seis meses) até 46 anos (mediana 12 anos), e de trabalho em centro cirúrgico entre zero (seis meses) e 40 anos (mediana de oito anos). Nos hospitais privados, a idade dos participantes variou entre 23 e 72 anos, média de 39,2 anos (desvio-padrão=10,9), a maioria estava na faixa de 30 a 49 anos (59,7%), entre um e 40 anos de formatura (mediana de 10 anos) e de trabalho em centro cirúrgico de zero (seis meses) até 30 anos (mediana seis anos e seis meses) (dados não apresentados em tabela).

No período intraoperatório, com relação ao controle da temperatura da sala de operação, os resultados evidenciaram que tal prática era mais frequente nos hospitais privados (88,7%) na comparação com os públicos (58,3%), sendo a diferença significante (p<0,001). O monitoramento da temperatura corporal do paciente também era a prática mais frequente nas instituições privadas (35,4%) em comparação com as públicas (29,4%) (p=0,027).

O termômetro axilar foi o dispositivo mais utilizado para mensurar a temperatura corporal nas instituições públicas (53,5%) e o esofágico (34,5%) nas privadas, p=0,003. O anestesista foi o profissional mais indicado como responsável pelo monitoramento da temperatura corporal do paciente, em ambos os tipos de hospitais, p=0,014.

Na Tabela 2, os dados sobre o pré-aquecimento como medida para a prevenção da hipotermia foram apresentados.

Na Tabela 3, as medidas para a prevenção de hipotermia foram apresentadas. O aumento da temperatura da sala de operação foi uma medida cujos resultados apresentaram diferença significante entre os tipos de hospitais (p=0,003), sendo que tal prática era realizada com frequência maior nos hospitais públicos. Com relação ao uso de método ativo de aquecimento cutâneo entre os tipos de instituições, os resultados apresentaram diferença significante (p=0,009). O sistema de ar forçado aquecido foi o método ativo indicado com frequência maior em ambos os tipos de instituições.

A enfermagem foi a categoria profissional indicada com frequência maior para a implementação de método ativo de aquecimento cutâneo, 59,7% nos hospitais públicos e 75,8% nos privados, sendo a diferença significante (p=0,027).

Na Tabela 4, as dificuldades para a prevenção da hipotermia foram apresentadas.

**Tabela 2-:** Pré-aquecimento do paciente antes da indução anestésica, informado pelos enfermeiros de centro cirúrgico de hospitais públicos e privados. Ribeirão Preto, SP, Brasil, 2022

Pré-aquecimento	Público (n*=139)		Privado (n*=62)		p ^‡^
	n ^*^	% ^†^	n ^*^	% ^†^	
Realizado no ambiente cirúrgico					0,008
Sim	98	70,5	55	88,7	
Não	41	29,5	7	11,3	
Medidas adotadas ^§^					0,055
Método passivo	39	28,1	22	35,5	
Método ativo	45	32,4	24	38,7	
Método misto	12	8,6	7	11,3	
Nunca realiza	41	29,5	7	11,3	
Profissional responsável ^ǁ^					0,029
Anestesista	16	11,5	6	9,7	
Enfermagem	57	41,0	33	53,2	
Ambas as categorias profissionais	19	13,7	13	21,0	
Nunca realiza	41	29,5	7	11,3	

*n = Número absoluto; ^†^% = Porcentagem; ^‡^Teste Qui-quadrado de Pearson; ^§^Não informaram: dois para cada tipo de hospital; ^ǁ^Não informaram: seis para hospital público e três para o privado

**Tabela 3-:** Medidas para a prevenção da hipotermia, no período intraoperatório, informadas pelos enfermeiros de centro cirúrgico de hospitais públicos e privados. Ribeirão Preto, SP, Brasil, 2022

Variáveis	Público (n*=139)		Privado (n*=62)		p ^‡^
	n*	% ^†^	n*	% ^†^	
Aumentar a temperatura da sala de operação					0,003 ^§^
Sim	57	41,0	12	19,4	
Não	82	59,0	50	80,6	
Uso de método passivo de aquecimento cutâneo					0,212 ^§^
Sim	67	48,2	24	38,7	
Não	72	51,8	38	61,3	
Uso de método ativo de aquecimento cutâneo					0,062 ^§^
Sim	77	55,4	43	69,4	
Não	62	44,6	19	30,6	
Uso de método misto					0,873 ^§^
Sim	19	13,7	9	14,5	
Não	120	86,3	53	85,5	
Infusão de solução aquecida por via endovenosa					0,385 ^§^
Sim	67	48,2	34	54,8	
Não	72	51,8	28	45,2	
Solução aquecida para lavagem de cavidades					0,285 ^§^
Sim	41	29,5	23	37,1	
Não	98	70,5	39	62,9	
Método ativo de aquecimento é realizado					0,009 ^ǁ^
Sim	113	81,3	59	95,2	
Não	26	18,7	3	4,8	
Profissional que realiza método ativo de aquecimento					
Anestesista					0,067 ^§^
Sim	68	48,9	39	62,9	
Não	71	51,1	23	37,1	
Cirurgião					0,403 ^§^
Sim	7	5,0	5	8,1	
Não	132	95,0	57	91,9	
Enfermagem					0,027 ^§^
Sim	83	59,7	47	75,8	
Não	56	40,3	15	24,2	

*n = Número absoluto; ^†^% = Porcentagem; ^‡^Nível de significância; ^§^Teste Qui-quadrado de Pearson; ^ǁ^Teste exato de Fisher

**Tabela 4-:** Dificuldades para a prevenção de hipotermia, no período intraoperatório, informadas pelos enfermeiros de centro cirúrgico de hospitais públicos e privados. Ribeirão Preto, SP, Brasil, 2022

Variáveis	Público (n*=139)		Privado (n*=62)		p ^‡^
	n*	% ^†^	n*	% ^†^	
Motivos para não realizar monitorização da temperatura corporal					
Equipe anestésica não julga relevante a adoção desta prática no centro cirúrgico					0,342 ^§^
Sim	33	23,7	11	17,7	
Não	106	76,3	51	82,3	
Equipe cirúrgica não julga relevante a adoção desta prática no centro cirúrgico					0,672 ^§^
Sim	13	9,4	7	11,3	
Não	126	90,6	65	88,7	
Dificuldades para a implementação desta prática entre os profissionais de saúde					0,118 ^§^
Sim	39	28,1	11	17,7	
Não	100	71,9	51	82,3	
Dispositivos médicos necessários não são suficientes para a adoção desta prática					0,014 ^§^
Sim	43	30,9	9	14,5	
Não	96	69,1	53	85,5	
Materiais utilizados para o aquecimento passivo são suficientes para a demanda diária					0,096 ^ǁ^
Nunca	17	12,2	3	4,8	
Raramente	17	12,2	3	4,8	
Às vezes	18	12,9	5	8,1	
Frequentemente	33	23,8	19	30,6	
Sempre	54	38,9	32	51,7	
Dificuldades para o uso de método ativo de aquecimento cutâneo					
Não tem dispositivos médicos suficientes para a demanda diária					0,080 ^§^
Sim	56	40,3	17	27,4	
Não	83	59,7	45	72,6	
Não tem dispositivos médicos para pacientes com fatores de risco					0,151 ^§^
Sim	21	15,1	15	24,2	
Não	118	84,9	47	75,8	
Tem dispositivos médicos suficientes para a demanda diária, mas os profissionais não consideram prática relevante					0,352 ^ǁ^
Sim	11	7,9	2	3,2	
Não	128	92,1	60	96,8	
Dificuldade de articulação entre os profissionais de saúde envolvidos no cuidado					0,159 ^§^
Sim	40	28,8	12	19,4	
Não	99	71,2	50	80,6	

*n = Número absoluto; ^†^% = Porcentagem; ^‡^Nível de significância; ^§^Teste Qui-quadrado de Pearson; ^ǁ^Teste exato de Fisher; Nota: as variáveis “motivos para não realizar monitorização da temperatura corporal” e “dificuldades para o uso de método ativo de aquecimento cutâneo” são de múltiplas escolhas, logo a totalidade supera o valor amostral

## Discussão

Conforme já mencionado, a hipotermia perioperatória está associada a diferentes complicações. Na literatura existem corpo de evidências e diretrizes clínicas de diferentes sociedades científicas, indicando a relevância na implementação de medidas para a prevenção desse evento, entretanto, na prática clínica, a ocorrência de hipotermia ainda é alta^(12-13)^.

No presente estudo, o controle da temperatura na sala de operação e o monitoramento da temperatura corporal do paciente foram práticas mais frequentes nos hospitais privados. Além disso, o termômetro axilar foi o dispositivo mais utilizado para mensurar a temperatura corporal nas instituições públicas e o esofágico em instituições privadas (todos os resultados com diferença estatística).

A temperatura da sala de operação é considerada como fator de risco para hipotermia perioperatória. Atualmente, o parâmetro recomendável é ≥ 21°C^(7,10,14)^. No perioperatório, em dois estudos observacionais, os autores investigaram práticas de monitoramento da temperatura corporal, sendo que em uma pesquisa (n=1.690 pacientes), o padrão de monitoramento foi baixo (n=687; 40,7%); no período pré-operatório (dentro de uma hora antes do paciente ser transferido para a sala de operação) e no período intraoperatório, somente 22,4% (n=379) dos participantes tiveram a temperatura monitorada ≥ uma vez, enquanto 94% (n=1.515) foram avaliados na admissão para os cuidados no pós-operatório. Os termômetros para o canal auditivo (infravermelho) e cavidade oral foram os mais frequentemente utilizados em todas as fases^(15)^.

No outro estudo, com a participação da equipe perioperatória (enfermeiros, anestesistas e pessoal técnico da anestesia), os resultados evidenciaram que a admissão para a cirurgia (n=384, 76%) consistiu na fase mais rotineira para monitorização da temperatura; no período intraoperatório, 56% dos entrevistados (n=281) responderam que o monitoramento da temperatura era realizado continuamente em seu local de trabalho e 93% (n=472) relataram o monitoramento da temperatura na chegada à sala de recuperação pós-anestésica. Os entrevistados identificaram que os dispositivos de canal auditivo são os mais utilizados tanto no pré-operatório (n=239, 47%) quanto no pós-operatório (n=286, 57%). No período intraoperatório, os dispositivos nasofaríngeos foram identificados como os mais frequentemente utilizados (n=331, 66%), seguido pelos esofágicos (n=229, 45%) e da bexiga urinária (n=202, 40%)^(12)^.

Em estudo longitudinal nacional, no período intraoperatório, os autores investigaram a confiabilidade das medições de temperatura corporal por meio de três métodos (termômetro temporal infravermelho periférico, termômetro cutâneo central, Zero-Heat-Flux, e termômetro esofágico ou nasofaríngeo). A amostra foi composta de 99 pacientes submetidos a cirurgias eletivas de câncer abdominal. Pautados nos resultados, os pesquisadores não recomendaram o uso de termômetro temporal infravermelho para mensurar a temperatura corporal. Os dois termômetros centrais testados foram equivalentes na detecção de hipotermia^(16)^.

Frente ao exposto, o uso do termômetro axilar para mensurar a temperatura corporal do paciente consiste em prática que deve ser repensada, principalmente nas instituições públicas.

O pré-aquecimento como medida para prevenção da hipotermia também era executado com frequência maior nos hospitais privados (resultados com diferença significante). Na literatura existe corpo de evidências que comprova a efetividade dessa medida para manter a normotermia do paciente cirúrgico. Em ensaio clínico randomizado, o objetivo delimitado foi avaliar o efeito de diferentes períodos de pré-aquecimento na temperatura corporal em pacientes submetidos à ressecção transuretral da bexiga ou próstata com anestesia geral. A amostra foi composta de 297 pacientes aleatorizadas em quatro grupos, a saber: grupo controle (n=76), grupo intervenção 15 minutos (n=74), grupo intervenção 30 minutos (n=73) e grupo intervenção 45 minutos (n=74). O pré-aquecimento foi realizado na sala pré-anestésica com o uso do sistema de ar forçado aquecido. No período intraoperatório, todos os participantes do estudo foram aquecidos também com o sistema de ar forçado aquecido. A temperatura central foi significativamente maior nos grupos de intervenção 15 e 30 minutos (36,8°C, p=0,004; 36,7°C, p=0,041, respectivamente). Além disso, no final da cirurgia, a temperatura corporal foi significativamente menor no grupo controle (35,8°C) do que nos três grupos que receberam o pré-aquecimento (p<0,001)^(17)^.

Em outro ensaio clínico randomizado, os autores investigaram o efeito do pré-aquecimento na temperatura corporal de pacientes submetidos à cirurgia abdominal (abordagem cirúrgica aberta) e o nível de conforto térmico. Os pacientes foram aleatorizados em três grupos: grupo controle (n=33; pré-aquecimento com cobertor e lençol de algodão); grupo intervenção 1 (n=33, pré-aquecimento com sistema de ar forçado aquecido por 20 minutos) e grupo intervenção 2 (n=33, pré-aquecimento com sistema de ar forçado aquecido por 30 minutos). No período intraoperatório, todos os pacientes foram aquecidos por meio do uso do sistema de ar forçado, além do aquecimento de fluidos de infusão e irrigação por equipamento específico. O conforto térmico foi determinado por meio de autorrelato do paciente nos períodos pré e pós-anestésico. A intervenção 2 apresentou os resultados melhores em relação à temperatura, com a média menor de episódios de temperatura abaixo de 36°C durante o período intraoperatório e conforto térmico maior relatado pelos pacientes^(18)^.

Em revisão sistemática com metanálise, os autores investigaram o efeito do pré-aquecimento (antes da indução anestésica) na temperatura corporal, com a inclusão de 27 ensaios clínicos randomizados. O tamanho da amostra dos estudos variou de 16 a 416 pacientes. Diferentes métodos ativos de aquecimento cutâneo foram empregados nos estudos (por exemplo, sistema de ar forçado aquecido, a cobertura elétrica, cobertura de fibra de carbono, entre outros), bem como diferentes tempos de aquecimento (10 a 120 minutos). Os resultados evidenciaram que o pré-aquecimento é medida efetiva para manutenção da normotermia perioperatória^(3)^.

Na presente pesquisa, nos dois tipos de hospitais, o sistema de ar forçado aquecido foi o método ativo indicado com frequência maior. Em revisão sistemática com metanálise, os autores investigaram a efetividade de diferentes sistemas de aquecimento ativo (sistema de ar forçado aquecido, sistema de circulação de água aquecida e cobertura elétrica) na prevenção da hipotermia e dos tremores quando aplicados em áreas específicas do corpo humano. Na revisão foram incluídos 24 ensaios clínicos randomizados, com a participação de 1.119 pacientes adultos submetidos a cirurgias abdominais. Os pacientes do grupo controle foram submetidos a métodos passivos de aquecimento. O desfecho primário mensurado foi a temperatura corporal central aos 60 e 120 minutos após a indução anestésica. Os diferentes dispositivos descartáveis (membros superiores, membros inferiores ou corpo todo colocado sobre a mesa de operação) do sistema de ar forçado foram investigados. Os resultados da metanálise em rede evidenciaram que o sistema de ar forçado (dispositivo corpo todo) efetivamente elevou a temperatura corporal central e evitou tremores dos pacientes em recuperação^(13)^.

Em outra revisão sistemática com metanálise, os pesquisadores também analisaram o efeito no uso do sistema de ar forçado aquecido e o uso de fluidos intravenosos aquecidos (estratégia combinada) em pacientes submetidas à cesárea com anestesia regional. Na revisão foram avaliados nove ensaios clínicos randomizados, com a participação de 595 mulheres. A estratégia combinada reduziu a incidência de hipotermia e tremores, com melhora no escore de conforto materno (resultados com diferença significante)^(19)^.

Em ensaio clínico randomizado, os autores investigaram o efeito do sistema de ar forçado aquecido, colocado em diferentes partes do corpo. Assim, 537 pacientes submetidos à cirurgia abdominal aberta foram randomizados nos grupos A (dispositivo descartável em membros superiores), B (membros inferiores) e C (corpo todo colocado sobre a mesa de operação). A hipotermia intraoperatória ocorreu em 51,4% dos pacientes do grupo B; 37,6% do grupo A e 34,1% do grupo C (p=0,002). Os resultados evidenciaram que o uso do sistema de ar forçado aquecido, colocado nos membros superiores ou corpo todo diminuiu a hipotermia e prolongou o tempo de manutenção da temperatura central acima de 36°C antes da instalação do evento^(20)^.

A enfermagem foi a categoria profissional responsável com frequência maior para a implementação do pré-aquecimento e de método ativo de aquecimento, em ambos os tipos de hospitais (resultados com diferença significante). O enfermeiro desempenha papel importante na prevenção de complicações do paciente cirúrgico, inclusive na prevenção de hipotermia, entretanto, a manutenção da normotermia é prática de responsabilidade de toda equipe perioperatória (cirurgiões, anestesistas e equipe de enfermagem)^(10,21)^.

Na presente pesquisa, entre os motivos para não realizar a monitorização da temperatura corporal, no período intraoperatório, apenas houve diferença significante entre os hospitais públicos e privados, o motivo foi que os dispositivos médicos necessários não são suficientes para a adoção da prática.

Em estudo qualitativo, os pesquisadores aplicaram um questionário *online*, para identificar os principais fatores que impedem os anestesistas na realização de manejo adequado da temperatura corporal. A amostra foi composta com 195 participantes, sendo que 81% (n=158) responderam que não conheciam as diretrizes clínicas para o monitoramento da temperatura corporal e, 95,4% (n=186) afirmaram às vezes negligenciar a realização de medidas para a prevenção da hipotermia, principalmente, durante cirurgia de emergência e transferências de pacientes. Duas categorias (ambiental e insuficiência de recursos) que dificultavam o monitoramento da temperatura foram relatadas por 99% (n=193) dos anestesistas. Apenas 12,8% (n=25) relataram que monitoravam frequentemente a temperatura corporal e realizavam isso como rotina diária^(22)^.

Em outro estudo qualitativo, o objetivo delimitado foi revelar as percepções de enfermeiros de centro cirúrgico (n=17) sobre a hipotermia perioperatória, bem como as experiências e recomendações para sua prevenção. A ausência de equipamentos, especificamente do sistema de ar forçado aquecido, devido à disponibilidade inadequada também foi uma barreira apontada no manejo deste evento. Os enfermeiros relataram que, na observação do paciente com frio, medidas alternativas foram implementadas como o uso de aquecedor de calor radiante e cobertura elétrica, a elevação da temperatura do ambiente ou o emprego de cobertura adicional (por exemplo, lençol de algodão aquecido). Além disso, o aquecimento de fluidos de irrigação e intravenosos, bem como sangue e hemoderivados também foram medidas relatadas pelos participantes da pesquisa. Os autores concluíram que as diferentes medidas mencionadas se devem à falta de padronização institucional, à inadequação do quantitativo de sistema ativo de aquecimento e à falta de protocolos de cuidados para orientar os enfermeiros no planejamento e na implementação de medidas para a prevenção da hipotermia^(23)^.

Apesar do corpo de evidências sobre a importância da prevenção da hipotermia perioperatória por meio da implementação de medidas efetivas; ainda existem lacunas entre a produção de conhecimento e a prática clínica. Tal cenário pode ocorrer devido ao conhecimento insuficiente da problemática pelos profissionais de saúde, bem como a baixa adesão destes no uso das diretrizes clínicas de sociedades científicas. Em estudo transversal, com a participação de enfermeiros e técnicos de anestesia (n=219), a pontuação média de conhecimento do profissional de saúde em relação ao manejo da hipotermia foi de 13,78, ou seja, 79,5% dos participantes apresentaram nível moderado (≤10 pontos: baixo; 11-20 pontos: moderado, ≥21 pontos: alto)^(10)^.

Em estudo tranversal, conduzido na China, o objetivo delimitado foi investigar o conhecimento, atitudes e práticas dos profissionais de saúde sobre a prevenção da hipotermia. A amostra foi composta por 14 cirurgiões, 29 anestesistas e 170 enfermeiros. As pontuações médias para conhecimento, atitudes e práticas foram de 5,36 (pontuação total de 12), 47,54 (pontuação total de 55) e 31,57 (pontuação total de 40), respectivamente. A maioria dos participantes apresentou atitudes positivas e práticas aceitáveis, entretanto, o conhecimento sobre a prevenção desse evento foi inadequado. Os autores destacaram a necessidade de programas de treinamento e protocolos padronizados para melhorar a adesão às diretrizes de prevenção da hipotermia perioperatória^(24)^.

Em outro estudo transversal, a amostra foi composta somente por enfermeiros (n=413). Os resultados evidenciaram que 59,1% dos participantes apresentaram bom conhecimento e a maioria (50,4%) tinha boas práticas sobre prevenção da hipotermia perioperatória. Os fatores associados ao conhecimento dos enfermeiros incluíram sexo masculino, ser bacharel, ter mestrado e participar de treinamento. Os fatores associados à prática clínica foram: trabalhar na sala de recuperação pós-anestésica ou unidade de terapia intensiva, participar de treinamento, estar satisfeito com o emprego e ter bom conhecimento. Os autores concluíram que os gestores hospitalares devem investir em estratégias (por exemplo, programas de treinamento) para incrementar o conhecimento e a implementação de medidas para a prevenção da hipotermia^(8)^.

Em estudo descritivo-exploratório nacional, o objetivo delimitado foi avaliar o conhecimento e intervenções da equipe de enfermagem sobre hipotermia perioperatória. Houve a participação de 77 membros da equipe de enfermagem (enfermeiros e técnicos) com atuação em centro cirúrgico e sala de recuperação pós-anestésica. Dos resultados evidenciados, 45,7% dos participantes relataram a importância no monitoramento da temperatura corporal durante o pré, intra e pós-operatório, sendo as seguintes práticas mais conhecidas: uso de aquecedor (35,5%), uso do sistema de ar forçado aquecido (28,3%), soro aquecido (16,9%) e cobertores (15,1%). Aquecer o paciente (58,6%) e administrar soro aquecido (31,5%) foram as duas medidas que obtiveram frequências maiores no centro cirúrgico. Os autores concluíram que ainda existem lacunas no conhecimento e nas práticas para a prevenção da hipotermia, sendo necessário o planejamento de ações educativas para incrementar a qualidade do cuidado prestado ao paciente cirúrgico^(25)^.

Em estudo descritivo, os pesquisadores também investigaram o conhecimento e práticas para a prevenção de hipotermia. A amostra foi composta de 122 enfermeiros. A elaboração do roteiro de coleta de dados foi pautada nas diretrizes clínicas da *Association of Perioperative Registered Nurses*; *National Institute for Health and Care Excellence*; *Turkish Society of Anesthesiology and Reanimation Guideline*. Os resultados demonstraram que 80,3% dos enfermeiros indicaram o uso de método passivo, 49,1% a utilização de método ativo (ar aquecido) e 36,9% o aquecimento de sangue e fluidos antes da administração. Na conclusão, os autores apontaram que os enfermeiros não utilizavam os métodos preconizados pelas sociedades científicas na prevenção da hipotermia perioperatória^(9)^.

Atualmente, na literatura existem recomendações gerais, pautadas em evidências, as quais podem nortear a construção e implementação de protocolos de cuidados para a prevenção de hipotermia perioperatória. Para todas as fases do perioperatório (pré, intra e pós), os principais princípios são a monitorização da temperatura corporal central, o aquecimento do paciente por método ativo e a redução da exposição ao frio. A monitorização da temperatura deve ser realizada para todos os pacientes e sempre que possível de forma contínua. O uso de método ativo de aquecimento deve ser adotado para manter a temperatura do paciente acima de 36^o^C e o seu conforto térmico (o sistema de ar forçado é recomendado). A exposição ao frio deve ser minimizada, mantendo sempre que possível o paciente coberto, evitando a perda de calor por radiação, condução, convecção e evaporação. No período intraoperatório, a temperatura da sala deve ser mantida em pelo menos 21^o^C, bem como implementar o uso de aquecimento de fluidos intravenosos^(5,26)^.

No perioperatório, a elaboração de um protocolo de cuidados para a prevenção da hipotermia pode auxiliar os profissionais de saúde na tomada de decisão sobre as melhores práticas para a monitorização da temperatura corporal e a implementação de medidas efetivas para a redução dessa complicação na prática clínica. Entretanto, a construção do protocolo deve estar em consonância com a compreensão das principais dificuldades vivenciadas no serviço de saúde para a manutenção da normotermia do paciente, por exemplo, considerando o quantitativo insuficiente de dispositivos tanto para a monitorização da temperatura corporal quanto para o uso de sistema ativo de aquecimento cutâneo. Além disso, a sensibilização reduzida dos gestores sobre a problemática pode inviabilizar os investimentos financeiros necessários em tecnologias^(4-5,10,27)^.

Frente aos resultados evidenciados, pode-se inferir que as medidas relatadas pelos enfermeiros para a prevenção da hipotermia estão em consonância com o que é preconizado na literatura. Em contrapartida, ainda existe a necessidade de incrementar a realização diária dessas medidas, principalmente nos hospitais públicos, uma vez que os percentuais relativos ao monitoramento da temperatura corporal do paciente, realização do pré-aquecimento e uso de método ativo de aquecimento cutâneo foram maiores nas instituições privadas (resultados com diferença significante). Além disso, há uma demanda de investimentos e qualificação de recursos humanos para o manejo efetivo deste evento comum no período intraoperatório.

A condução do estudo gerou corpo de evidências que pode subsidiar a prática clínica e auxiliar a tomada de decisão dos profissionais de saúde envolvidos no cuidado do paciente cirúrgico, em especial o enfermeiro perioperatório. A prevenção da hipotermia pode reduzir as complicações associadas a este evento, melhorar os resultados para o paciente e, principalmente, incrementar as boas práticas na saúde.

Com relação às limitações da pesquisa, salienta-se uma que se refere ao tipo de estudo, ou seja, os dados apresentados e discutidos foram relatados pelos participantes. Apesar de o pesquisador principal impulsionar sistematicamente o convite para os enfermeiros, via redes sociais e correio eletrônico, o tamanho da amostra poderia ser maior, o que pode ser caracterizado como baixa adesão dos respondentes.

## Conclusão

O sistema de ar forçado aquecido foi o método ativo que obteve frequência maior e a enfermagem, a categoria profissional mais indicada como responsável pela implementação do pré-aquecimento e uso de método ativo, em hospitais públicos e privados.

No período intraoperatório, manter a normotermia do paciente ainda é um desafio, pois exige trabalho conjunto entre cirurgiões, anestesistas e equipe de enfermagem. Acrescido a esta situação, as instituições de saúde precisam investir na capacitação dos profissionais de saúde envolvidos no cuidado do paciente cirúrgico, na aquisição de dispositivos médicos adequados para a monitorização da temperatura corporal e na compra de equipamentos efetivos de aquecimento cutâneo.

## Data Availability

O conjunto de dados que dá suporte aos resultados deste estudo não está disponível publicamente.

# Translation: Prevención de la hipotermia en el periodo intraoperatorio: estudio descriptivo-exploratorio*

**DOI:** 10.1590/1518-8345.7694.4727

**Authors:** Layze Braz de Oliveira, Vanessa de Brito Poveda, Rosana Aparecida Spadoti Dantas, Julio Cesar Ribeiro, Cristina Maria Galvão

**Affiliations:** 1 Centro Universitário UniFacid, Instituto de Educação Médica, Teresina, PI, Brasil. Centro Universitário UniFacid Instituto de Educação Médica PI Teresina Brasil; 2 Becaria de la Coordenação de Aperfeiçoamento de Pessoal de Nível Superior (CAPES), Brasil. Becaria de la Coordenação de Aperfeiçoamento de Pessoal de Nível Superior Brasil; 3 Universidade de São Paulo, Centro Colaborador de la OPS/OMS para el Desarrollo de la Investigación en Enfermería, Ribeirão Preto, SP, Brasil. Universidade de São Paulo Centro Colaborador de la OPS/OMS para el Desarrollo de la Investigación en Enfermería SP Ribeirão Preto Brasil; 4 Universidade de Franca, Faculdade de Enfermagem, Franca, SP, Brasil. Universidade de Franca Faculdade de Enfermagem SP Franca Brasil; 5 Becaria del Conselho Nacional de Desenvolvimento Científico e Tecnológico (CNPq), Brasil. Becaria del Conselho Nacional de Desenvolvimento Científico e Tecnológico Brasil

**Keywords:** Enfermería Perioperatoria, Hipotermia, Periodo Intraoperatorio, Temperatura Corporal, Hospitales, Seguridad del Paciente

## Abstract

analizar las medidas implementadas en la práctica para la prevención de la hipotermia durante el periodo intraoperatorio en hospitales públicos y privados.

estudio cuantitativo, descriptivo-exploratorio. La muestra estuvo compuesta por 201 enfermeros, invitados por intermedio de redes sociales, *Facebook* e *Instagram*, y correo electrónico. La recolección de datos se realizó mediante un formulario en línea creado en la plataforma virtual *Survey Monkey*^®^. Para el análisis se aplicaron estadística descriptiva y pruebas de asociación con el fin de investigar las diferencias entre los grupos delimitados en relación con las variables del estudio.

en ambos tipos de instituciones, la mayoría de los participantes era de sexo femenino y estaba casada o en unión estable. Se observó una diferencia significativa entre los tipos de hospitales en el control de la temperatura del quirófano (p<0,001), el monitoreo de la temperatura corporal del paciente (p=0,027) y el uso de un método activo de calentamiento cutáneo (p=0,009)

el sistema de aire forzado calentado fue el método activo con mayor frecuencia de uso, y la enfermería fue la categoría profesional más señalada como responsable de la implementación del precalentamiento y del método activo en los tipos de instituciones investigadas. Aún es necesario incrementar la ejecución diaria de medidas para la prevención de la hipotermia, lo que señala a los gestores la necesidad de invertir en recursos y en la capacitación del personal.

## Destacados

**(1)** La hipotermia perioperatoria es un evento común que puede afectar al paciente quirúrgico. **(2)** La prevención de la hipotermia es una práctica desafiante para todo el equipo perioperatorio. **(3)** La medición de la temperatura corporal es una práctica recomendada durante el periodo intraoperatorio. **(4)** El precalentamiento es una medida relevante para el mantenimiento de la normotermia. **(5)** El sistema de aire forzado calentado es una medida eficaz para la prevención de la hipotermia.

## Introducción

La hipotermia perioperatoria se define comúnmente como una temperatura corporal por debajo de 36°C y está relacionada, principalmente, con el uso de agentes anestésicos que provocan alteraciones en la regulación hipotalámica de la temperatura y en la redistribución del calor corporal. Otros factores que pueden contribuir a este suceso incluyen la baja temperatura ambiental, la exposición de órganos o tejidos del paciente, la temperatura ambiente de las soluciones intravenosas y de los fluidos de irrigación^(1-3)^.

En la literatura existe un cuerpo de evidencias que indica que la hipotermia perioperatoria se asocia a eventos cardíacos, infección de sitio quirúrgico, aumento de la pérdida sanguínea y de la necesidad de transfusión, disminución del metabolismo de medicamentos, malestar térmico, aumento de la permanencia en la sala de recuperación posanestésica y de los costos hospitalarios^(1-5)^.

En la práctica clínica, la ocurrencia de esta complicación es común. En Estados Unidos, en el perioperatorio, la incidencia de hipotermia varió entre 20% y 70%^(3)^ y, en Australia, fue del 42%^(4)^. En Brasil, su prevalencia fue del 56,7%^(6)^.

En una revisión sistemática con metaanálisis, los autores investigaron los factores de riesgo de hipotermia durante el periodo intraoperatorio en pacientes adultos. La muestra estuvo compuesta por 12 estudios que involucraron a 15.010 pacientes. La edad, el índice de masa corporal, la temperatura ambiental, la presión arterial sistólica y la frecuencia cardíaca (en el periodo preoperatorio), la duración prolongada de la anestesia (> 2 horas) y la administración de soluciones intravenosas (> 1.000 ml) fueron señalados como factores de riesgo^(7)^.

Las principales recomendaciones para la prevención de la hipotermia perioperatoria se orientan al monitoreo y documentación de la temperatura corporal del paciente en el perioperatorio, al uso de métodos activos para el mantenimiento de la normotermia (por ejemplo, el sistema de aire forzado calentado), incluida la implementación del precalentamiento antes de la inducción anestésica cuando la temperatura del paciente sea inferior a 36°C. También se recomienda la combinación de métodos pasivos (por ejemplo, el uso de sábana de algodón calentada) con activos, así como evaluar los factores específicos relacionados con el paciente (por ejemplo, la edad) y aquellos relacionados con el procedimiento anestésico-quirúrgico (por ejemplo, tipo y duración de la cirugía y de la anestesia). Además, minimizar la exposición del paciente a un ambiente frío es una estrategia para reducir la pérdida de calor. Otro aspecto relevante es mantener la temperatura ambiental, recomendándose al menos 21°C^(2,5)^.

A pesar de la relevancia de la prevención de la hipotermia perioperatoria destacada en la literatura, en la práctica clínica se registran problemas en el reconocimiento de la importancia de este evento por parte de los profesionales de la salud y de los gestores de las instituciones, en el monitoreo de la temperatura corporal del paciente y en la implementación de métodos activos. Además, el conocimiento y la práctica de los enfermeros sobre la problemática son inadecuados en comparación con las directrices clínicas (*guidelines*) de las sociedades científicas^(8-10)^.

Frente a lo expuesto, el presente estudio se condujo para generar un cuerpo de evidencias que sustente la práctica clínica, dado que el mantenimiento de la temperatura corporal del paciente y la implementación de medidas efectivas para la prevención de la hipotermia en el perioperatorio siguen siendo retos para el equipo de profesionales de la salud que atiende al paciente quirúrgico, en especial el enfermero. Además, se destaca la escasez de estudios sobre la problemática en el escenario nacional, con énfasis en instituciones de diferente naturaleza jurídica. Así, el objetivo delimitado fue analizar las medidas implementadas en la práctica para la prevención de la hipotermia durante el periodo intraoperatorio en hospitales públicos y privados.

## Método

### Dseño del estudio

Se trata de un estudio cuantitativo, descriptivo-exploratorio.

### Lugar

El lugar de recolección de datos no se determinó por tratarse de una investigación realizada en un entorno virtual, en el cual los posibles participantes podían ser incluidos en el estudio independientemente de su ubicación física en todo el territorio nacional.

### Población y criterios de elegibilidad de la muestra

La población objetivo del estudio estuvo compuesta por enfermeros que trabajaban en centros quirúrgicos (CC) de hospitales brasileños, públicos y privados. Se conformó una muestra no probabilística de 201 enfermeros que cumplían los siguientes criterios de elegibilidad: poseer vínculo laboral activo como enfermero, desempeñarse en el CC de la institución hospitalaria durante al menos seis meses, sin distinción del tipo de actividades realizadas, y acceder al formulario en línea entre enero y agosto de 2022. Siete enfermeros que completaron íntegramente el formulario no integraron la muestra final por trabajar como perfusionistas (tres) y en la central de material esterilizado (dos), además de dos que cursaban residencia en clínica quirúrgica. Un técnico de enfermería respondió a la encuesta y tampoco fue considerado.

### Recolección de datos

Para el reclutamiento en línea de los posibles participantes se emplearon diferentes estrategias en las redes sociales *Facebook* e *Instagram*. En *Facebook* se identificó el grupo “Enfermería en centro quirúrgico y centro de material esterilizado”, que cuenta con 1,8 mil miembros. En dicho grupo se identificaron los potenciales participantes, es decir, enfermeros que trabajaban en CC, y el investigador principal impulsó una publicación dirigida a las personas seleccionadas mediante criterios como: lugar de trabajo, personas que habían indicado “Me gusta” en la página del investigador, así como sus amistades y públicos personalizados definidos previamente.

Al consultar el *Instagram* de la *Associação Brasileira de Enfermeiros de Centro Cirúrgico, Recuperação Anestésica e Centro de Material e Esterilização* (SOBECC), el investigador principal envió una invitación al perfil de cada profesional que indicaba en su biografía formación en enfermería con actuación en CC.

La SOBECC dispone de una base de correos electrónicos de enfermeros que actúan en el cuidado del paciente en el perioperatorio. Tras la aprobación de la junta directiva de la organización para utilizar dicha base, el investigador principal envió a los miembros registrados el enlace electrónico generado por la plataforma virtual *Survey Monkey*^®^. Debido a la baja adhesión de los participantes, se realizaron dos intentos mediante esta estrategia de recolección de datos.

A los posibles participantes identificados se facilitó un enlace electrónico que brindaba acceso, entre enero y agosto de 2022, a la invitación del estudio, al Consentimiento Informado (CI) y al formulario de recolección de datos.

### Formulario de recopilación de datos y variables del estudio

Se creó un formulario en línea en la plataforma virtual *Survey Monkey*^®^, con 28 preguntas divididas en tres secciones: nueve para la caracterización de los enfermeros, 16 sobre las medidas para la prevención de la hipotermia durante el periodo intraoperatorio y tres sobre las dificultades para prevenir esta complicación. Las preguntas se elaboraron con base en la literatura^(2,11)^ y en la producción intelectual de los autores del presente estudio sobre la problemática investigada.

La variable dependiente fue el tipo de institución de salud (pública o privada). Las variables independientes se agruparon por temas, a saber: caracterización sociodemográfica (por ejemplo: sexo, edad, estado civil) y de formación de los enfermeros (por ejemplo: tipo de formación complementaria), medidas para la prevención de la hipotermia (por ejemplo: uso de precalentamiento, métodos activos y pasivos) y dificultades para la prevención de este evento durante el periodo intraoperatorio (por ejemplo: motivos para no realizar el monitoreo de la temperatura corporal del paciente y la disponibilidad de dispositivos médicos).

### Tratamiento y análisis de los datos

Los datos se almacenaron en hojas de cálculo en formato *Excel* y, posteriormente, se transfirieron al *software* IBM^®^*SPSS*^®^*Statistics* versión 25.

Para la presentación de los datos, los participantes se dividieron en dos grupos: enfermeros que trabajaban en el CC de hospitales públicos y aquellos que lo hacían en instituciones privadas. Las variables cualitativas se describieron mediante la frecuencia (absoluta y relativa) de distribución de los participantes entre las categorías delimitadas, y las variables cuantitativas se evaluaron según la medida de posición (media) y dispersión (desviación estándar). Se aplicaron pruebas de asociación (prueba de chi-cuadrado de Pearson o prueba exacta de Fisher) para investigar las diferencias entre los grupos delimitados en relación con las variables medidas de precalentamiento, medidas para la prevención de la hipotermia y dificultades para prevenir este evento durante el periodo intraoperatorio. Para todos los análisis se consideró un nivel de significancia del 5% (α=0,05).

### Aspectos éticos

El estudio fue aprobado por el Comité de Ética en Investigación local, bajo dictamen CAAE: 43126521.5.0000.5393. Todos los participantes otorgaron el CI en modalidad en línea, mediante la selección de la opción “Acepto participar en la investigación”. Se respetaron todos los preceptos éticos establecidos en la legislación.

## Resultados

En la Tabla 1 se presentan las variables sociodemográficas y de formación de los participantes del estudio. En ambos tipos de hospital, la mayoría de los participantes era de sexo femenino, casada o en unión estable, con especialización y un único vínculo laboral.

**Tabla 1-:** Variables sociodemográficas y de formación de los enfermeros que trabajan en el centro quirúrgico de hospitales públicos y privados. Ribeirão Preto, SP, Brasil, 2022

Variables	Público (n*=139)		Privado (n*=62)		Total (n*=201)	
	n*	% ^†^	n*	% ^†^	n*	% ^†^
Sexo						
Femenino	119	85,6	56	90,3	175	87,1
Masculino	20	14,4	6	9,7	26	12,9
Rango etario (años)						
<30	11	7,9	13	21,0	24	11,9
30 – 39	58	41,7	24	38,7	82	40,8
40 – 49	48	34,5	13	21,0	61	30,3
50 – 59	13	9,4	8	12,9	21	10,4
≥60	9	6,5	4	6,5	13	6,5
Estado civil						
Soltero	46	33,1	23	37,1	69	34,3
Casado/unión estable	76	54,7	32	51,6	108	53,7
Divorciado/separado/viudo	17	12,2	7	11,3	24	12,0
Tiempo de formación (años)						
<5	15	10,8	14	22,6	29	14,4
5 – 10	33	23,7	18	29,0	51	25,4
>10	91	65,5	30	48,4	121	60,2
Formación complementaria						
Sí	122	87,8	55	88,7	177	88,1
No	17	12,2	7	11,3	24	11,9
Niveles de formación complementaria						
Residencia	2	1,4	1	1,6	3	1,5
Especialización	86	61,9	47	75,8	133	66,2
Maestría	21	15,1	4	6,5	25	12,4
Doctorado	13	9,4	2	3,2	15	7,5
No especificó	-	-	1	1,6	1	0,5
No posee	17	12,2	7	11,3	24	11,9
Vínculos laborales						
Uno	104	74,8	52	83,9	156	77,6
Dos	35	25,2	10	16,1	45	22,4
Tiempo de trabajo en el centro quirúrgico (años)						
<5	43	30,9	23	37,1	66	32,8
5 – 10	34	24,5	18	29,0	52	25,9
>10	62	44,6	21	33,9	83	41,3

*n = Número absoluto; ^†^% = Porcentaje

En los hospitales públicos, la edad de los enfermeros varió entre 23 y 74 años, con una media de 40,9 años (desviación estándar = 9,6), concentrándose en el rango de 30 a 49 años (76,2%), con tiempo de formación desde cero (seis meses) hasta 46 años (mediana de 12 años), y tiempo de trabajo en centro quirúrgico entre cero (seis meses) y 40 años (mediana de ocho años). En los hospitales privados, la edad de los participantes osciló entre 23 y 72 años, con una media de 39,2 años (desviación estándar = 10,9); la mayoría se encontraba en el rango de 30 a 49 años (59,7%), con entre uno y 40 años desde la graduación (mediana de 10 años) y tiempo de trabajo en centro quirúrgico de cero (seis meses) hasta 30 años (mediana de seis años y seis meses) (datos no presentados en tabla).

Durante el periodo intraoperatorio, en cuanto al control de la temperatura del quirófano, los resultados evidenciaron que esta práctica era más frecuente en los hospitales privados (88,7%) en comparación con los públicos (58,3%), con diferencia significativa (p<0,001). El monitoreo de la temperatura corporal del paciente también era más frecuente en las instituciones privadas (35,4%) que en las públicas (29,4%) (p=0,027).

El termómetro axilar fue el dispositivo más utilizado para medir la temperatura corporal en las instituciones públicas (53,5%) y el esofágico (34,5%) en las privadas, p=0,003. El anestesista fue el profesional más señalado como responsable del monitoreo de la temperatura corporal del paciente en ambos tipos de hospitales, p=0,014.

En la Tabla 2 se presentan los datos sobre el precalentamiento como medida para la prevención de la hipotermia.

En la Tabla 3 se presentan las medidas para la prevención de la hipotermia. El aumento de la temperatura del quirófano fue una medida cuyos resultados mostraron diferencia significativa entre los tipos de hospitales (p=0,003), siendo esta práctica realizada con mayor frecuencia en los hospitales públicos. En cuanto al uso de método activo de calentamiento cutáneo entre los tipos de instituciones, los resultados también presentaron diferencia significativa (p=0,009). El sistema de aire forzado calentado fue el método activo señalado con mayor frecuencia en ambos tipos de instituciones.

La enfermería fue la categoría profesional señalada con mayor frecuencia como responsable de la implementación del método activo de calentamiento cutáneo, 59,7% en los hospitales públicos y 75,8% en los privados, siendo la diferencia significativa (p=0,027).

En la Tabla 4 se presentan las dificultades para la prevención de la hipotermia.

**Tabla 2-:** Precalentamiento del paciente antes de la inducción anestésica, según lo informado por enfermeros del centro quirúrgico de hospitales públicos y privados. Ribeirão Preto, SP, Brasil, 2022

Precalentamiento	Público (n*=139)		Privado (n*=62)		p ^‡^
	n ^*^	% ^†^	n ^*^	% ^†^	
Realizado en el entorno quirúrgico					0,008
Sí	98	70,5	55	88,7	
No	41	29,5	7	11,3	
Medidas adoptadas ^§^					0,055
Método pasivo	39	28,1	22	35,5	
Método activo	45	32,4	24	38,7	
Método mixto	12	8,6	7	11,3	
Nunca se realiza	41	29,5	7	11,3	
Profesional responsable ^ǁ^					0,029
Anestesista	16	11,5	6	9,7	
Enfermería	57	41,0	33	53,2	
Ambas categorías profesionales	19	13,7	13	21,0	
Nunca se realiza	41	29,5	7	11,3	

*n = Número absoluto; ^†^% = Porcentaje; ^‡^Prueba de chi-cuadrado de Pearson; ^§^No informaron: dos para cada tipo de hospital; ^ǁ^No informaron: seis en hospital público y tres en el privado

**Tabla 3-:** Medidas para la prevención de la hipotermia durante el periodo intraoperatorio, informadas por enfermeros del centro quirúrgico de hospitales públicos y privados. Ribeirão Preto, SP, Brasil, 2022

Precalentamiento	Público (n*=139)		Privado (n*=62)		p ^‡^
	n ^*^	% ^†^	n ^*^	% ^†^	
Aumentar la temperatura del quirófano					0,003 ^§^
Sí	57	41,0	12	19,4	
No	82	59,0	50	80,6	
Uso de método pasivo de calentamiento cutáneo					0,212 ^§^
Sí	67	48,2	24	38,7	
No	72	51,8	38	61,3	
Uso de método activo de calentamiento cutáneo					0,062 ^§^
Sí	77	55,4	43	69,4	
No	62	44,6	19	30,6	
Uso de método mixto					0,873 ^§^
Sí	19	13,7	9	14,5	
No	120	86,3	53	85,5	
Infusión de solución calentada por vía intravenosa					0,385 ^§^
Sim	67	48,2	34	54,8	
No	72	51,8	28	45,2	
Solución calentada para lavado de cavidades					0,285 ^§^
Sí	41	29,5	23	37,1	
No	98	70,5	39	62,9	
Se realiza método activo de calentamiento					0,009 ^ǁ^
Sí	113	81,3	59	95,2	
No	26	18,7	3	4,8	
Profesional que realiza el método activo de calentamiento					
Anestesista					0,067 ^§^
Sí	68	48,9	39	62,9	
No	71	51,1	23	37,1	
Cirujano					0,403 ^§^
Sí	7	5,0	5	8,1	
No	132	95,0	57	91,9	
Enfermería					0,027 ^§^
Sí	83	59,7	47	75,8	
No	56	40,3	15	24,2	

*n = Número absoluto; ^†^% = Porcentaje; ^‡^Nivel de significancia; ^§^Prueba de chi-cuadrado de Pearson; ^ǁ^Prueba exacta de Fisher

**Tabla 4-:** Dificultades para la prevención de la hipotermia durante el periodo intraoperatorio, informadas por enfermeros del centro quirúrgico de hospitales públicos y privados. Ribeirão Preto, SP, Brasil, 2022

Precalentamiento	Público (n*=139)		Privado (n*=62)		p ^‡^
	n ^*^	% ^†^	n ^*^	% ^†^	
Motivos para no realizar el monitoreo de la temperatura corporal					
El equipo de anestesia no considera relevante la adopción de esta práctica en el centro quirúrgico					0,342 ^§^
Sí	33	23,7	11	17,7	
No	106	76,3	51	82,3	
El equipo quirúrgico no considera relevante la adopción de esta práctica en el centro quirúrgico					0,672 ^§^
Sí	13	9,4	7	11,3	
No	126	90,6	65	88,7	
Dificultades para la implementación de esta práctica entre los profesionales de la salud					0,118 ^§^
Sí	39	28,1	11	17,7	
No	100	71,9	51	82,3	
No hay suficientes dispositivos médicos para la adopción de esta práctica					0,014 ^§^
Sí	43	30,9	9	14,5	
No	96	69,1	53	85,5	
Materiales utilizados para el calentamiento pasivo son suficientes para la demanda diaria					0,096 ^ǁ^
Nunca	17	12,2	3	4,8	
Raramente	17	12,2	3	4,8	
A veces	18	12,9	5	8,1	
Frecuentemente	33	23,8	19	30,6	
Siempre	54	38,9	32	51,7	
Dificultades para el uso de método activo de calentamiento cutáneo					
No hay suficientes dispositivos médicos para la demanda diaria					0,080 ^§^
Sí	56	40,3	17	27,4	
No	83	59,7	45	72,6	
No hay dispositivos médicos para pacientes con factores de riesgo					0,151 ^§^
Sí	21	15,1	15	24,2	
No	118	84,9	47	75,8	
Hay suficientes dispositivos médicos para la demanda diaria, pero los profesionales no consideran relevante la práctica					0,352 ^ǁ^
Sí	11	7,9	2	3,2	
No	128	92,1	60	96,8	
Dificultad de articulación entre los profesionales de la salud involucrados en el cuidado					0,159 ^§^
Sí	40	28,8	12	19,4	
No	99	71,2	50	80,6	

*n = Número absoluto; ^†^% = Porcentaje; ^‡^Nivel de significancia; ^§^Prueba de chi-cuadrado de Pearson; ^ǁ^Prueba exacta de Fisher; Nota: las variables motivos para no realizar el monitoreo de la temperatura corporal y dificultades para el uso de método activo de calentamiento cutáneo son de selección múltiple, por lo tanto, el total supera el valor muestral

## Discusión

Como ya se mencionó, la hipotermia perioperatoria está asociada a diversas complicaciones. En la literatura existen cuerpos de evidencia y directrices clínicas de distintas sociedades científicas que indican la relevancia de implementar medidas para la prevención de este evento; sin embargo, en la práctica clínica, la ocurrencia de hipotermia sigue siendo elevada^(12-13)^.

En el presente estudio, el control de la temperatura en la sala de operaciones y el monitoreo de la temperatura corporal del paciente fueron prácticas más frecuentes en los hospitales privados. Además, el termómetro axilar fue el dispositivo más utilizado para medir la temperatura corporal en las instituciones públicas y el esofágico en instituciones privadas (todos los resultados con diferencia estadística).

La temperatura de la sala de operaciones es considerada un factor de riesgo para la hipotermia perioperatoria. Actualmente, el parámetro recomendable es ≥ 21ºC^(7,10,14)^. En el perioperatorio, en dos estudios observacionales, los autores investigaron prácticas de monitoreo de la temperatura corporal. En una investigación (n=1.690 pacientes), el estándar de monitoreo fue bajo (n=687; 40,7%); en el período preoperatorio (dentro de una hora antes de que el paciente fuera trasladado a la sala de operaciones) y en el período intraoperatorio, solo el 22,4% (n=379) de los participantes tuvo la temperatura monitoreada al menos una vez, mientras que el 94% (n=1.515) fue evaluado al ingreso a los cuidados postoperatorios. Los termómetros para el canal auditivo (infrarrojo) y la cavidad oral fueron los más frecuentemente utilizados en todas las fases^(15)^.

En otro estudio, con la participación del equipo perioperatorio (enfermeros, anestesistas y personal técnico de anestesia), los resultados evidenciaron que el ingreso a cirugía (n=384, 76%) fue la fase más rutinaria para el monitoreo de la temperatura; en el período intraoperatorio, el 56% de los entrevistados (n=281) respondió que el monitoreo de la temperatura se realizaba de forma continua en su lugar de trabajo y el 93% (n=472) reportó el monitoreo de la temperatura al ingreso a la sala de recuperación posanestésica. Los entrevistados identificaron que los dispositivos de canal auditivo son los más utilizados tanto en el preoperatorio (n=239, 47%) como en el postoperatorio (n=286, 57%). En el período intraoperatorio, los dispositivos nasofaríngeos fueron identificados como los más frecuentemente utilizados (n=331, 66%), seguidos por los esofágicos (n=229, 45%) y los de vejiga urinaria (n=202, 40%)^(12)^.

En un estudio longitudinal nacional, en el período intraoperatorio, los autores investigaron la confiabilidad de las mediciones de temperatura corporal mediante tres métodos (termómetro temporal infrarrojo periférico, termómetro cutáneo central, *Zero-Heat-Flux*, y termómetro esofágico o nasofaríngeo). La muestra estuvo compuesta por 99 pacientes sometidos a cirugías electivas de cáncer abdominal. Con base en los resultados, los investigadores no recomendaron el uso del termómetro temporal infrarrojo para medir la temperatura corporal. Los dos termómetros centrales evaluados fueron equivalentes en la detección de hipotermia^(16)^.

Ante lo expuesto, el uso del termómetro axilar para medir la temperatura corporal del paciente es una práctica que debe ser replanteada, principalmente en las instituciones públicas.

El precalentamiento como medida para la prevención de la hipotermia también se ejecutaba con mayor frecuencia en los hospitales privados (resultados con diferencia significativa). En la literatura existe un cuerpo de evidencia que comprueba la efectividad de esta medida para mantener la normotermia del paciente quirúrgico. En un ensayo clínico aleatorizado, el objetivo delimitado fue evaluar el efecto de diferentes períodos de precalentamiento sobre la temperatura corporal en pacientes sometidos a resección transuretral (vejiga o próstata) con anestesia general. La muestra estuvo compuesta por 297 pacientes aleatorizados en cuatro grupos: grupo control (n=76), grupo intervención 15 minutos (n=74), grupo intervención 30 minutos (n=73) y grupo intervención 45 minutos (n=74). El precalentamiento se realizó en la sala preanestésica utilizando el sistema de aire forzado calentado. En el período intraoperatorio, todos los participantes del estudio también fueron calentados con el sistema de aire forzado calentado. La temperatura central fue significativamente mayor en los grupos de intervención de 15 y 30 minutos (36,8°C, p=0,004; 36,7°C, p=0,041, respectivamente). Además, al final de la cirugía, la temperatura corporal fue significativamente menor en el grupo control (35,8°C) que en los tres grupos que recibieron el precalentamiento (p<0,001)^(17)^.

En otro ensayo clínico aleatorizado, los autores investigaron el efecto del precalentamiento sobre la temperatura corporal de pacientes sometidos a cirugía abdominal (abordaje quirúrgico abierto) y el nivel de confort térmico. Los pacientes fueron aleatorizados en tres grupos: grupo control (n=33; precalentamiento con manta y sábana de algodón); grupo intervención 1 (n=33, precalentamiento con sistema de aire forzado calentado durante 20 minutos) y grupo intervención 2 (n=33, precalentamiento con sistema de aire forzado calentado durante 30 minutos). En el período intraoperatorio, todos los pacientes fueron calentados mediante el uso del sistema de aire forzado, además del calentamiento de fluidos de infusión e irrigación con equipo específico. El confort térmico fue determinado mediante autorreporte del paciente en los períodos pre y posanestésico. La intervención 2 presentó los mejores resultados en relación con la temperatura, con el promedio menor de episodios de temperatura por debajo de 36°C durante el período intraoperatorio y mayor confort térmico reportado por los pacientes^(18)^.

En una revisión sistemática con metaanálisis, los autores investigaron el efecto del precalentamiento (antes de la inducción anestésica) sobre la temperatura corporal, con la inclusión de 27 ensayos clínicos aleatorizados. El tamaño de la muestra de los estudios varió de 16 a 416 pacientes. Se emplearon diferentes métodos activos de calentamiento cutáneo en los estudios (por ejemplo, sistema de aire forzado calentado, manta eléctrica, manta de fibra de carbono, entre otros), así como diferentes tiempos de calentamiento (10 a 120 minutos). Los resultados evidenciaron que el precalentamiento es una medida efectiva para el mantenimiento de la normotermia perioperatoria^(3)^.

En la presente investigación, en ambos tipos de hospitales, el sistema de aire forzado calentado fue el método activo indicado con mayor frecuencia. En una revisión sistemática con metaanálisis, los autores investigaron la efectividad de diferentes sistemas de calentamiento activo (sistema de aire forzado calentado, sistema de circulación de agua caliente y manta eléctrica) en la prevención de la hipotermia y los escalofríos cuando se aplicaban en áreas específicas del cuerpo humano. En la revisión se incluyeron 24 ensayos clínicos aleatorizados, con la participación de 1.119 pacientes adultos sometidos a cirugías abdominales. Los pacientes del grupo control fueron sometidos a métodos pasivos de calentamiento. El desenlace primario medido fue la temperatura corporal central a los 60 y 120 minutos después de la inducción anestésica. Se investigaron los diferentes dispositivos desechables (miembros superiores, miembros inferiores o cuerpo completo colocado sobre la mesa de operaciones) del sistema de aire forzado. Los resultados del metaanálisis en red evidenciaron que el sistema de aire forzado (dispositivo cuerpo completo) elevó efectivamente la temperatura corporal central y evitó los escalofríos de los pacientes en recuperación^(13)^.

En otra revisión sistemática con metaanálisis, los investigadores también analizaron el efecto del uso del sistema de aire forzado calentado y el uso de fluidos intravenosos calentados (estrategia combinada) en pacientes sometidas a cesárea con anestesia regional. En la revisión se evaluaron nueve ensayos clínicos aleatorizados, con la participación de 595 mujeres. La estrategia combinada redujo la incidencia de hipotermia y escalofríos, con mejora en la puntuación de confort materno (resultados con diferencia significativa)^(19)^.

En un ensayo clínico aleatorizado, los autores investigaron el efecto del sistema de aire forzado calentado, colocado en diferentes partes del cuerpo. Así, 537 pacientes sometidos a cirugía abdominal abierta fueron aleatorizados en los grupos A (dispositivo desechable en miembros superiores), B (miembros inferiores) y C (cuerpo completo colocado sobre la mesa de operaciones). La hipotermia intraoperatoria ocurrió en el 51,4% de los pacientes del grupo B; 37,6% del grupo A y 34,1% del grupo C (p=0,002). Los resultados evidenciaron que el uso del sistema de aire forzado calentado, colocado en los miembros superiores o cuerpo completo disminuyó la hipotermia y prolongó el tiempo de mantenimiento de la temperatura central por encima de 36°C antes de la instalación de este evento^(20)^.

La enfermería fue la categoría profesional responsable con mayor frecuencia de la implementación del precalentamiento y del método activo de calentamiento, en ambos tipos de hospitales (resultados con diferencia significativa). El enfermero desempeña un papel importante en la prevención de complicaciones del paciente quirúrgico, incluida la prevención de la hipotermia; sin embargo, el mantenimiento de la normotermia es una práctica de responsabilidad de todo el equipo perioperatorio (cirujanos, anestesistas y equipo de enfermería)^(10,21)^.

En la presente investigación, entre los motivos para no realizar el monitoreo de la temperatura corporal en el período intraoperatorio, solo hubo diferencia significativa entre los hospitales públicos y privados, siendo el motivo que los dispositivos médicos necesarios no son suficientes para la adopción de esta práctica.

En un estudio cualitativo, los investigadores aplicaron un cuestionario online para identificar los principales factores que impiden a los anestesistas realizar un manejo adecuado de la temperatura corporal. La muestra estuvo compuesta por 195 participantes, siendo que el 81% (n=158) respondió que no conocía las directrices clínicas para el monitoreo de la temperatura corporal y el 95,4% (n=186) afirmó que a veces descuidaba la realización de medidas para la prevención de la hipotermia, principalmente durante cirugías de emergencia y traslados de pacientes. Dos categorías (ambiental e insuficiencia de recursos) que dificultaban el monitoreo de la temperatura fueron reportadas por el 99% (n=193) de los anestesistas. Solo el 12,8% (n=25) relató que monitoreaba frecuentemente la temperatura corporal y lo hacía como rutina diaria^(22)^.

En otro estudio cualitativo, el objetivo delimitado fue revelar las percepciones de enfermeros de centro quirúrgico (n=17) sobre la hipotermia perioperatoria, así como sus experiencias y recomendaciones para su prevención. La ausencia de equipos, específicamente del sistema de aire forzado calentado, debido a la disponibilidad inadecuada, también fue una barrera señalada en el manejo de este evento. Los enfermeros relataron que, al observar al paciente con frío, se implementaron medidas alternativas como el uso de calefactores de calor radiante y cobertores eléctricos, el aumento de la temperatura del ambiente o el uso de cobertores adicionales (por ejemplo, sábanas de algodón calentadas). Además, el calentamiento de fluidos de irrigación e intravenosos, así como sangre y hemoderivados, también fueron medidas mencionadas por los participantes del estudio. Los autores concluyeron que las diferentes medidas mencionadas se deben a la falta de estandarización institucional, a la inadecuación de la cantidad de sistemas activos de calentamiento y a la ausencia de protocolos de cuidado que orienten a los enfermeros en la planificación e implementación de medidas para la prevención de la hipotermia^(23)^.

A pesar del cuerpo de evidencias sobre la importancia de la prevención de la hipotermia perioperatoria mediante la implementación de medidas efectivas, aún existen brechas entre la producción de conocimiento y la práctica clínica. Tal escenario puede ocurrir debido al conocimiento insuficiente de esta problemática por parte de los profesionales de salud, así como a la baja adhesión de estos al uso de las directrices clínicas de sociedades científicas. En un estudio transversal, con la participación de enfermeros y técnicos en anestesia (n=219), la puntuación media de conocimiento del profesional de salud respecto al manejo de la hipotermia fue de 13,78, es decir, el 79,5% de los participantes presentó un nivel moderado (≤10 puntos: bajo; 11-20 puntos: moderado; ≥21 puntos: alto)^(10)^.

En un estudio transversal, conducido en China, el objetivo delimitado fue investigar el conocimiento, actitudes y prácticas de los profesionales de salud sobre la prevención de la hipotermia. La muestra estuvo compuesta por 14 cirujanos, 29 anestesistas y 170 enfermeros. Las puntuaciones medias para conocimiento, actitudes y prácticas fueron de 5,36 (puntuación total de 12), 47,54 (puntuación total de 55) y 31,57 (puntuación total de 40), respectivamente. La mayoría de los participantes presentó actitudes positivas y prácticas aceptables, sin embargo, el conocimiento sobre la prevención de este evento fue inadecuado. Los autores destacaron la necesidad de programas de entrenamiento y protocolos estandarizados para mejorar la adhesión a las directrices de prevención de la hipotermia perioperatoria^(24)^.

En otro estudio transversal, la muestra estuvo compuesta únicamente por enfermeros (n=413). Los resultados evidenciaron que el 59,1% de los participantes presentó buen conocimiento y la mayoría (50,4%) tenía buenas prácticas sobre prevención de la hipotermia perioperatoria. Los factores asociados al conocimiento de los enfermeros incluyeron sexo masculino, ser licenciado, tener maestría y participar en entrenamiento. Los factores asociados a la práctica clínica fueron trabajar en la sala de recuperación posanestésica o unidad de cuidados intensivos, participar en entrenamiento, estar satisfecho con el empleo y tener buen conocimiento. Los autores concluyeron que los gestores hospitalarios deben invertir en estrategias (por ejemplo, programas de entrenamiento) para incrementar el conocimiento y la implementación de medidas para la prevención de la hipotermia^(8)^.

En un estudio descriptivo-exploratorio nacional, el objetivo delimitado fue evaluar el conocimiento e intervenciones del equipo de enfermería sobre la hipotermia perioperatoria. Participaron 77 miembros del equipo de enfermería (enfermeros y técnicos) con actuación en centro quirúrgico y sala de recuperación posanestésica. De los resultados evidenciados, el 45,7% de los participantes relató la importancia del monitoreo de la temperatura corporal durante el pre, intra y posoperatorio, siendo las siguientes prácticas las más conocidas: uso de calefactor (35,5%), uso del sistema de aire forzado calentado (28,3%), suero calentado (16,9%) y cobertores (15,1%). Calentar al paciente (58,6%) y administrar suero calentado (31,5%) fueron las dos medidas que obtuvieron mayores frecuencias en el centro quirúrgico. Los autores concluyeron que aún existen brechas en el conocimiento y prácticas para la prevención de la hipotermia, siendo necesario el planeamiento de acciones educativas para incrementar la calidad del cuidado prestado al paciente quirúrgico^(25)^.

En un estudio descriptivo, los investigadores también investigaron el conocimiento y prácticas para la prevención de la hipotermia. La muestra estuvo compuesta por 122 enfermeros. La elaboración del guion de recolección de datos se basó en las directrices clínicas de la *Association of Perioperative Registered Nurses*, *National Institute for Health and Care Excellence* y *Turkish Society of Anesthesiology and Reanimation Guideline*. Los resultados demostraron que el 80,3% de los enfermeros indicó el uso de método pasivo, el 49,1% la utilización de método activo (aire calentado) y el 36,9% el calentamiento de sangre y fluidos antes de la administración. En la conclusión, los autores señalaron que los enfermeros no utilizaban los métodos recomendados por las sociedades científicas en la prevención de la hipotermia perioperatoria^(9)^.

Actualmente, en la literatura existen recomendaciones generales, basadas en evidencia, que pueden orientar la construcción e implementación de protocolos de cuidados para la prevención de la hipotermia perioperatoria. Para todas las fases del perioperatorio (pre, intra y postoperatorio), los principales principios son la monitorización de la temperatura corporal central, el calentamiento del paciente mediante un método activo y la reducción de la exposición al frío. La monitorización de la temperatura debe realizarse en todos los pacientes y, siempre que sea posible, de forma continua. El uso de un método activo de calentamiento debe adoptarse para mantener la temperatura del paciente por encima de los 36°C y garantizar su confort térmico (se recomienda el sistema de aire forzado calentado). La exposición al frío debe minimizarse, manteniendo al paciente cubierto siempre que sea posible y evitando la pérdida de calor por radiación, conducción, convección y evaporación. Durante el período intraoperatorio, la temperatura de la sala debe mantenerse en al menos 21°C, así como debe implementarse el calentamiento de los fluidos intravenosos^(5,26)^.

En el perioperatorio, la elaboración de un protocolo de cuidados para la prevención de la hipotermia puede ayudar a los profesionales de la salud en la toma de decisiones sobre las mejores prácticas para la monitorización de la temperatura corporal y la implementación de medidas efectivas para reducir esta complicación en la práctica clínica. Sin embargo, la construcción del protocolo debe estar alineada con la comprensión de las principales dificultades vividas en el servicio de salud para mantener la normotermia del paciente, como por ejemplo, la cantidad insuficiente de dispositivos tanto para la monitorización de la temperatura corporal como para el uso del sistema activo de calentamiento cutáneo. Además, la escasa sensibilización de los gestores respecto a esta problemática puede inviabilizar las inversiones financieras necesarias en tecnologías^(4-5,10,27)^.

Frente a los resultados evidenciados, se puede inferir que las medidas relatadas por los enfermeros para la prevención de la hipotermia están en consonancia con lo que se recomienda en la literatura. Por otro lado, aún es necesario incrementar la realización diaria de estas medidas, principalmente en los hospitales públicos, dado que los porcentajes relativos al monitoreo de la temperatura corporal del paciente, la realización del precalentamiento y el uso de métodos activos de calentamiento cutáneo fueron mayores en las instituciones privadas (resultados con diferencia significativa). Además, se destaca la necesidad de inversiones y de cualificación de los recursos humanos para el manejo efectivo de este evento común en el período intraoperatorio.

La realización del estudio generó un cuerpo de evidencia que puede respaldar la práctica clínica y auxiliar la toma de decisiones de los profesionales de salud involucrados en el cuidado del paciente quirúrgico, especialmente el enfermero perioperatorio. La prevención de la hipotermia puede reducir las complicaciones asociadas a este evento, mejorar los resultados para el paciente y, principalmente, fortalecer las buenas prácticas en salud.

En cuanto a las limitaciones del estudio, se destaca una relacionada con el tipo de estudio, es decir, los datos presentados y discutidos fueron autorreportados por los participantes. A pesar de que el investigador principal promovió sistemáticamente la invitación a los enfermeros a través de redes sociales y correo electrónico, el tamaño de la muestra podría haber sido mayor, lo que puede caracterizarse como una baja adhesión por parte de los encuestados.

## Conclusión

El sistema de aire forzado calentado fue el método activo que presentó mayor frecuencia de uso, y la enfermería fue la categoría profesional más señalada como responsable por la implementación del precalentamiento y del uso de métodos activos, en hospitales públicos y privados.

Durante el período intraoperatorio, mantener la normotermia del paciente sigue siendo un desafío, ya que exige un trabajo conjunto entre cirujanos, anestesistas y el equipo de enfermería. Además, las instituciones de salud necesitan invertir en la capacitación de los profesionales involucrados en el cuidado del paciente quirúrgico, en la adquisición de dispositivos médicos adecuados para la monitorización de la temperatura corporal y en la compra de equipos efectivos de calentamiento cutáneo.

## Data Availability

El conjunto de datos que respalda los resultados de este estudio no está disponible públicamente.
